# Examining disparities in harmful reporting on community firearm violence in Philadelphia television news reports

**DOI:** 10.1186/s40621-026-00659-4

**Published:** 2026-02-01

**Authors:** Jessica H. Beard, Evan L. Eschliman, Jennifer Midberry, Leah E. Roberts, Anita Wamakima, Kallie Palm, Siena Wanders, Tyrone Muns, Jim MacMillan, Christopher N. Morrison

**Affiliations:** 1 Division of Trauma Surgery and Surgical Critical Care, Department of Surgery, Temple University Hospital, Philadelphia, PA USA; 2 Lewis Katz School of Medicine at Temple University, Philadelphia, PA USA; 3 Department of Epidemiology, Mailman School of Public Health, Columbia University, New York, NY USA; 4 Klein College of Media and Communication, Temple University, Philadelphia, PA USA; 5 Temple University Hospital, Philadelphia, PA USA; 6 Philadelphia Center for Gun Violence Reporting, Philadelphia, PA USA; 7 Department of Epidemiology and Preventive Medicine, School of Public Health and Preventive Medicine, Monash University, Clayton, VIC Australia

**Keywords:** Firearm violence, Firearm injury, Framing, Media, News reporting, Journalism

## Abstract

**Background:**

News coverage of community firearm violence (CFV) can cause multilevel harm. We aimed to quantify the frequency and severity of harmful CFV news content and examine how victim, shooting event, and place-based characteristics relate to harmful CFV reporting.

**Methods:**

We performed a quantitative media content analysis of a random sample of CFV clips aired on Philadelphia local television news in 2021 using a novel codebook. We matched clips to shooting victims in the Philadelphia Police Department dataset and used shooting event location to obtain place-based characteristics from American Community Survey data. Generalized structural equation models evaluated associations between victim demographics, shooting event, and place-based characteristics (Group I), news coverage characteristics (Group II), and either composite harmful CFV reporting scores or specific harmful reporting elements determined by a prior modified Delphi process (Group III).

**Results:**

Among 394 individuals covered in 303 clips, the mean (SD) individual-level harmful CFV reporting score was 15.2 (4.38) out of 29. Episodic framing (i.e. reporting lacking systematic context) (93.9%) was the most common harmful content element and perpetrator mugshot (4.8%) was least common element. Graphic and/or explicit content was present in 30.3% of clips. Individuals having more than one clip and longer total focus time had higher harmful reporting scores, while individuals involved in fatal shootings, having longer total clip length, and having a follow-up story had lower scores. Black, adult, victims in non-fatal shootings and people shot in areas with a higher proportion of Black residents were more likely to have news coverage containing graphic and/or explicit content.

**Conclusions:**

Harmful CFV reporting is pervasive, and the severity of harm is associated with news coverage characteristics. Disparities in specific harmful CFV content elements may further exacerbate existing health inequities. Journalists should modify CFV reporting practices to minimize harm.

**Supplementary Information:**

The online version contains supplementary material available at 10.1186/s40621-026-00659-4.

## Background

Firearm-related injury remains a significant threat to public health in the United States (US) [1–3]. Community firearm violence (CFV), which includes fatal and non-fatal shootings that result from interpersonal violence, disproportionately harms communities that have been marginalized by historic and ongoing political disinvestment [4–8]. Research indicates that many common aspects of CFV news coverage can deepen existing inequities by shaping how the public and policy makers perceive and respond to firearm-related injury [9–13]. For example, despite a number of evidence-based public health approaches to address CFV, US news outlets rarely contextualize CFV within its root causes and solutions [12, 14–17]. Instead, news reports on violence tend to highlight individual culpability instead of structural causes and emphasize policing over public health responses to CFV [12, 14, 18–20]. There are many gaps in our understanding of reporting on CFV, including how news coverage contributes to CFV prevention and harm, how CFV reporting differs by salient characteristics (e.g., victim, perpetrator, location, time), and what constitutes best practices for journalists covering CFV to minimize harm.

Our previous research found that Philadelphia, PA, television news systematically overreported shootings of children, mass shootings, and shootings that occurred in neighborhoods with higher median household income, less socioeconomic inequality and lower degrees of racialized economic segregation [21]. Other studies have found similar reporting disparities, estimating that only about half of shooting victims make the news in Philadelphia, PA, Rochester, NY, and Cincinnati, OH [22]. Implications for systematic reporting biases are significant, as an inaccurate public understanding of firearm violence can lead to inappropriate allocation of resources, including research funding [21, 23]. Public health researchers have urged journalists to report on common firearm-related risks, while media organizations recommend providing epidemiologic context in CFV news reports [23, 24].

News coverage of CFV can be harmful across socioecological levels (e.g., individual, community, and society) [25, 26]. One way that journalists and news networks transmit messages that may lead to multilevel harm is through *framing* [27]. Framing in news occurs when journalists determine which content to include or exclude in a story, whose perspectives to feature, and which event characteristics to highlight [27]. These decisions make certain aspects of a story more salient to audiences, and news consumers are likely to internalize these perspectives when they are repeated in news over time [27–29]. *Episodic crime* framing is news framing that focuses on a specific shooting event in isolation, defines violence as a crime issue, and privileges police narrators above other perspectives. This type of framing can lead news audiences to blame victims, reinforce racialized narratives about communities impacted by violence, and undermine effective public health responses [12, 13, 17, 30]. In our previous research, firearm injury survivors described how episodic crime narratives in news reports of their shootings made them feel dehumanized and compounded their trauma [31]. Firearm-injured people also described how specific news content elements, including graphic content, factual inaccuracies, and naming the treating hospital resulted in distress, harms to reputation, and threats to personal safety [31]. At the community level, some participants in the study expressed beliefs that harmful reporting may be driving increased CFV incidence in their neighborhoods by generating fear, which could increase firearm purchasing and carrying [31]. Existing evidence indicates that harmful CFV reporting is a threat to health across multiple levels; therefore, mitigating this harm is an important target to minimize individual-level trauma and support effective societal responses to CFV.

Journalists aim to minimize harm to victims and news audiences while they seek truth and report the news [32]. As researchers and advocates have pointed out harmful reporting practices in recent decades, journalistic guidelines have evolved in response, providing special instructions on covering suicide, mass shootings, sexual assault, abuse, and crime involving minors [32, 33]. There is a clear precedent of research on harmful news content leading to public health interventions in the form of modified news reporting protocols. For example, studies demonstrating that harmful reporting on suicide is associated with increases in suicide incidence led to the creation and implementation of journalistic guidelines for suicide reporting endorsed by public health experts [34–37]. These guidelines include specific harm-reduction recommendations for news outlets to avoid, including: prominent story placement, sensationalized content, oversimplification of suicide, discussion of the suicide method, and repeated reporting on the same event [36, 37]. When journalists follow these guidelines to limit harmful content, portray suicide as preventable, and provide resources, population-level decreases in suicide rates have been observed [34, 38, 39].

Until recently, conceptualizations of harmful CFV reporting were far less developed than those that exist for coverage of suicide and mass shootings. To fill this gap, our team used a modified three-round Delphi process to achieve a consensus definition of harmful CFV reporting [25]. Twenty-one experts from three expertise groups (lived experience of CFV, journalism practice, and scholarship) participated in all three rounds of the study. The process yielded a definition specifying the 12 content elements that comprise potentially harmful CFV news reporting along severity ratings of harm (mild, moderate, severe) caused by each element at three socioecological levels (individual, community, and society) [25]. These 12 content elements are displayed in Table 1, along with their proposed extent of potential harm (i.e., severe, moderate, or mild) at each socioecological level of harm (i.e., individual, community, or society). Delphi panel experts determined that CFV news stories which include graphic content, use episodic framing, and fail to explore solutions have the potential to cause severe harm at all three levels [25]. In addition, panelists decided that the harmful news content elements were most detrimental to firearm-injured people, with 8 of 12 elements rated as severe harm at the individual level.

**Table 1:** Harmful CFV reporting content elements identified via expert consensus and ratings of harm at three socioecological levels

Harmful community firearm violence reporting content elements	Expert consensus of severity of harm at each level		
	Individual	Community	Society
Does not explore solutions	Severe	Severe	Severe
Graphic and/or explicit content	Severe	Severe	Severe
Overall episodic framing	Severe	Severe	Severe
Only law enforcement narrators	Severe	Moderate	Severe
Absence of a follow-up story	Severe	Moderate	Moderate
Missing perspective of firearm-injured person	Severe	Moderate	Moderate
Missing community perspective	Severe	Moderate	Moderate
Name of treating hospital	Severe	Moderate	Mild
Mugshot of perpetrator	Moderate	Moderate	Moderate
Number of gunshot wounds sustained by the firearm-injured person	Moderate	Moderate	Moderate
Relationship between firearm-injured person and perpetrator	Moderate	Moderate	Moderate
Clinical condition of firearm-injured person	Moderate	Moderate	Mild

The current study extends our prior work by using a novel instrument and scale based on the expert consensus achieved in the prior modified Delphi process in order to: (1) quantify the frequency and severity of harmful CFV news content in Philadelphia television (TV) news coverage of CFV, and (2) examine associations between victim, shooting event, place-based, and news coverage-related characteristics and the composite harmful CFV reporting score as well as specific harmful CFV reporting elements. The goal of this research is to further establish an evidence base for the multilevel harm of existing CFV reporting paradigms by examining disparities in harmful CFV reporting. Our results will ultimately inform interventions to reduce harmful CFV reporting.

## Methods

### Data and data sources

#### TV news content

To generate data on CFV TV news content, we conducted a quantitative media content analysis on a random sample of TV news reports drawn from a database previously compiled by our team. Television is the news modality with the largest reach that also creates original reporting, making it an ideal source of data for CFV news content analysis [40, 41]. The creation of this database has been described in detail elsewhere [14, 21, 42]. In brief, we used TVEyes, a subscription TV news monitoring platform, to compile 7,497 TV news clips about firearm violence during three daily newscasts (6AM, 6PM, 11PM) on the four Philadelphia local English-language TV news stations (6ABC, CBS3, NBC10, and FOX29) in 2021. For this study sample, we randomly selected a broadcast time and news station for each day, then retained all clips from those times and stations that mentioned a specific shooting victim shot in Philadelphia County. This process was modeled after best practices for sampling in broadcast media content analysis [43] and resulted in a total of 323 clips.

For the quantitative media content analysis, we developed a novel instrument, the *Harmful Reporting Codebook*, to measure the 12 harmful CFV news content elements identified through expert consensus discussed previously (see **Appendix A** for the complete codebook) [25]. Three of the 12 harmful content elements required special consideration during codebook development and data analysis. “Overall episodic framing” was determined by whether the majority of the clip focused on a specific shooting event(s) and was in contrast to thematic framing, in which clips mostly discussed firearm violence more broadly, including social context, epidemiological trends, root causes, and/or solutions [14, 15]. The element of “only law enforcement narrators” was determined to be present if [1] no one apart from the journalist was featured in the clip and the journalist attributed information to only police sources, or [2] a law enforcement representative was the only narrator featured apart from the journalist. Finally, instead of the twelfth harmful reporting content element, “absence of a follow-up story,” clips were coded as whether or not they were a follow-up story. This was done as our sampling methodology did not allow us to determine if a follow-up story existed for each news report.

In addition to the information necessary for determining the presence of harmful content elements, the codebook contains variables for clip length (in seconds), shooting date, shooting location, and shooting time, along with variables that describe the shooting event and victim(s). These include whether the shooting was fatal or non-fatal and the demographic characteristics of the victim(s). The victim-level variables were included to facilitate the eventual matching of shooting victim and event data with the Philadelphia Police Department dataset.

Prior to coding the sample of clips, a team of four coders (TM, KP, AW, SW) supervised by a journalism researcher (JM) coded practice clips (outside the study sample) in three rounds, iterating the codebook and its instructions until reaching intercoder reliability (ICR; measured using Gwet’s AC_1_) for each variable of at least 0.700 [43]. The variable with the lowest percent agreement and Gwet’s AC_1_ in Round 3 of coding practice was whether or not the clip was a follow-up (Percent agreement: 88.5%, Gwet’s AC_1_ of 0.786). See **Appendix B** for a complete table of variables with ICR scores. These same coders then coded an overlapping 10% of the study sample clips, meeting the 0.700 Gwet’s AC_1_ ICR threshold. From then on, the remaining clips were divided among the four coders and single coded.

#### Philadelphia police department data

We then matched our clips to shootings in the publicly available Philadelphia Police Department (PPD) “Shooting Victims” dataset to collect shooting victim demographic information and shooting event characteristics [44]. This dataset is the most comprehensive epidemiologic data source for CFV in the city, and provides person-level reports of shootings that result from interpersonal violence in Philadelphia, comprising police-reported sex, age, and race/ethnicity of person injured; shooting outcome for that individual (i.e., fatality, injury); shooting date; shooting time; and block level shooting event location. Prior to matching these data to our clips, entries in this dataset were aggregated to the shooting event based on shooting time (i.e., within one hour), shooting location (i.e., within 100 m), and/or concordance in the PPD dataset identifiers (i.e., some person-level entries from the same event used the same identifier) according to a previously described methodology [45]. One co-author (LER) then manually matched news reports to the event(s) included in the PPD dataset by comparing the date, time, and description of individual(s) injured, matching all but 35 clips to a shooting victim/event in the PPD dataset. The unmatched clips were reviewed by three co-authors (JB, AW, SW), and all but 20 clips were able to be matched with the PPD data. The PPD research team assisted with a final round of matching, leaving just four news clips unmatched to PPD data. Two of those clips were excluded from analysis and two were retained as the information about the victims and shooting event were complete in the retained clips. Finally, one news clip was excluded because the event did not occur during 2021, leaving up to 320 clips for analysis.

#### American community survey data

Then, to finalize construction of the analytic dataset, the latitude and longitude from the PPD dataset was used to determine the census block group of each shooting event. Using census block group, we then joined American Community Survey 5-year 2015–2019 census block group-level and census tract-level estimates of socio-structural characteristics (e.g., population race/ethnicity, socioeconomic conditions, and social disadvantage) that are theoretically related to CFV incidence and severity and frequency of harmful CFV reporting [4, 6, 21, 46]. Because of data suppression rules, the American Community Survey 5-year 2015–2019 estimates were not available for the locations of shootings experienced by 22 individuals and covered by 17 clips.

### Measures

#### Dependent variables

The main dependent variables were harmful CFV reporting scores at the individual, community, and society levels and the presence of each of 11 harmful reporting elements (i.e., “absence of follow-up story” was not included as a harmful reporting element in this analysis due to methodological limitations described above) across any of the clips about each shooting event. We determined the harmful CFV reporting scores by assigning values to each of the 11 elements (0 if present, then 1, 2, or 3 if rated as having mild, moderate, or severe harm, respectively) using Delphi panelists’ ratings of the severity of its harm at each level, then summing the values [25]. We elected to utilize a summed score for harm by level to allow for comparison of harm severity across clips. The maximum possible scores were 29 for the individual level, 25 for the community level, and 24 for the society level. These maximum scores were disparate because Delphi panelists rated harm for each element slightly differently across levels of harm (see Table 1). For determining individual harmful reporting elements, we considered all the clips covering each shooting event such that any of the clips containing one element meant that event’s news reporting had that element. For the four elements that referenced an absence of content (i.e., “only law enforcement narrators,” “missing perspective of firearm-injured person,” “missing community perspective,” and “does not cover solutions”), these had to occur across all clips covering the shooting event for that event’s news reporting to contain that element. Harmful CFV reporting scores were modeled as continuous and the presence of individual elements as binary.

#### Independent variables

We had two levels of independent variables. The first level comprised demographic characteristics of the individuals injured or killed in the shootings covered in our sample of clips (i.e., police-reported race/ethnicity, age recoded to “under 18” and “18 and older,” and sex), shooting event characteristics (i.e., whether the shooting involved a fatality, whether the shooting was a mass shooting defined as injuring four or more people), and place-based characteristics of where the shooting took place at the level of either census block group (i.e., percentage of Black residents, percentage of unemployment) or census tract (i.e., median household income, percentage of residents with income below the Census Bureau poverty threshold, income inequality measured using the Gini coefficient, and racialized economic segregation measured using the Index of Concentration at the Extremes (ICE) [47]). Notably, while the victim characteristics were specific to each individual, the same event and place-based characteristics were applied to all individuals in the same shooting (i.e., who thus have the same clip or set of clips as any other individual in the same shooting). The second level comprised variables conceptually related to the general characteristics of the news coverage we observed for each individual: whether there was more than one clip, total clip length (i.e., the sum of all clip lengths for each event), total focus time (i.e., the sum of the length of time clips are focused on a specific shooting event), and whether the coverage included a follow-up story. Like the event and place-based characteristics, individuals in the same shooting have the same coverage characteristics. Victim and shooting event characteristics were modeled as categorical or binary, while data on all place-based characteristics were used to create quintiles and modeled as continuous. For coverage characteristics, more than one clip and whether the coverage included a follow-up story was modeled as binary, while total clip length and total focus time were used to create quintiles and modeled as continuous. More detail on all variables, their data sources, and how they are modeled is provided in **Appendix C**.

### Statistical analysis

All analyses were completed using only individuals with complete data, meaning the 22 individuals whose shooting locations did not have all publicly available American Community Survey estimates were excluded, along with 17 corresponding clips. We used descriptive statistics to summarize the victim, shooting event, place-based, and coverage characteristics for individuals included in the final dataset. To measure the severity and frequency of harmful CFV reporting, we determined the median and interquartile range (IQR) of the harmful CFV reporting score for each of the three socioecological levels (individual, community, and society), and tabulated the presence of each harmful reporting element. To explore relationships between the presence and absence of the elements, we calculated pairwise correlations.

**Fig. 1:**
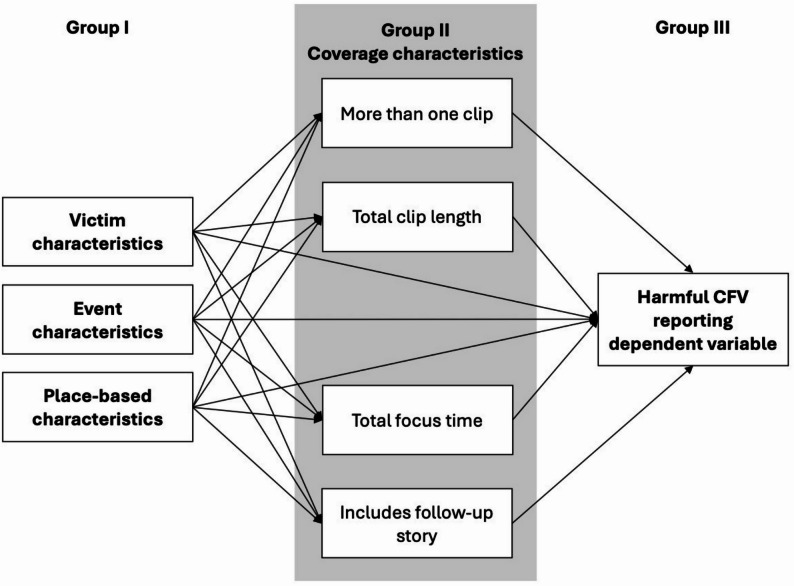
Specification of generalized structural equation models relating victim, event, and place-based characteristics to coverage characteristics and harmful community firearm violence reporting

Because we hypothesized that victim, shooting event, and place-based characteristics would influence both the general characteristics of CFV coverage and the inclusion of specific harmful content elements, and that the coverage characteristics themselves could pattern the presence of harmful content elements, we utilized generalized structural equation models (GSEM) with maximum likelihood estimation to evaluate the associations between victim, shooting event, and place-based characteristics (Group I), coverage characteristics (Group II), and each harmful reporting variable (i.e., either composite scores or each element) (Group III). Paths were specified from all variables in Group I to Group II and Group III as well as from all variables in Group II to Group III. Figure 1 displays the specification used for each model. We did not specify separate one-element models for any harmful reporting element that had a frequency of > 0.90.

We used Stata Version 19.5 for all analyses. In our reporting of results, we provide unstandardized estimates and draw attention to estimates with *p* < .05, *p* < .01, and *p* < .001. The [hidden for peer review] University Institutional Review Board determined that this analysis of publicly available TV news clips was not human subjects research and did not require review.

## Results

### Descriptive statistics

Our sample of 303 news clips included coverage of 394 firearm-injured people. Table 2 displays the victim, shooting event, place-based, and coverage characteristics for individuals included in the analyzed clips. The majority (83.4%) of these individuals had their race/ethnicity documented by police as Black, with 8.9% as white, 7.2% as Hispanic, and 0.5% as Asian. The median age of these individuals was 25 years old (IQR: 18–33), and a majority (80.0%) were adults (18 years and older). Most individuals (83.7%) had their sex documented by police as male. For shooting event characteristics, 40.1% of individuals were involved in a fatal shooting, and 13.7% were involved in a mass shooting. Individuals were shot in areas that had mean (SD) 60.8% (33.2) Black residents, 11.4% (11.4) of residents unemployed at the census block group level, median household income of $41,046.71 (24,470.63), 30.7% (13.5) residents with income under the Census Bureau poverty threshold, 0.47 (0.07) Gini coefficient, and − 0.08 (0.15) ICE scores for racialized economic segregation at the census tract level.

**Table 2:** Victim, event, place-based, and coverage characteristics for individuals included in coverage analyzed (*N* = 394)

**Victim characteristics**	
Race/ethnicity, *n* (%)	
Black	330 (83.8)
Hispanic	28 (7.1)
Asian	2 (0.5)
White	34 (8.6)
Age, *n* (%)	
18 and older	315 (79.9)
Under 18	73 (20.1)
Sex, *n* (%)	
Male	329 (83.5)
Female	65 (16.5)
**Event characteristics**	
Fatal shooting, *n* (%)	
Non-fatal	185 (46.9)
Fatal	209 (53.1)
Mass shooting, *n* (%)	
Not a mass shooting	343 (87.1)
Mass shooting	51 (12.9)
**Place-based characteristics**	
Percent Black residents, CBG, mean (SD)	60.1 (33.4)
Percent of residents unemployed, CBG, mean (SD)	11.4 (11.3)
Median household income, $USD, CT, mean (SD)	40,412 (22,850)
Percent of residents with income under federal poverty line, CT, mean (SD)	31.1 (13.3)
Income inequality, CT, mean (SD)	0.47 (0.07)
Racialized economic segregation, CT, mean (SD)	-0.07 (0.15)
**Coverage characteristics**	
Number of clips, *n* (%)	
One clip	301 (76.4)
More than one clip	93 (23.6)
Total clip length, seconds, mean (SD)	87.7 (91.9)
Total focus time, seconds, mean (SD)	67.0 (74.3)
Follow-up story, *n* (%)	
Does not include a follow-up story	229 (58.1)
Includes a follow-up story	165 (41.9)

CBG = census block group; CT = census tract; GSEM = generalized structural equation model

For coverage characteristics, the number of clips ranged from 1 to 5, and 24.3% of individuals were involved in shootings covered by more than one clip. The mean (SD) number of clips per individual was 1.31 (0.66). The mean (SD) total clip length was 89.0 (93.0) seconds, with total clip length ranging from 10 to 663 s. The mean (SD) total focus time was 67.6 (74.6) seconds, with total focus time ranging from 0 to 639 s. 42.1% of individuals had coverage that included at least one follow-up story.

**Fig. 2:**
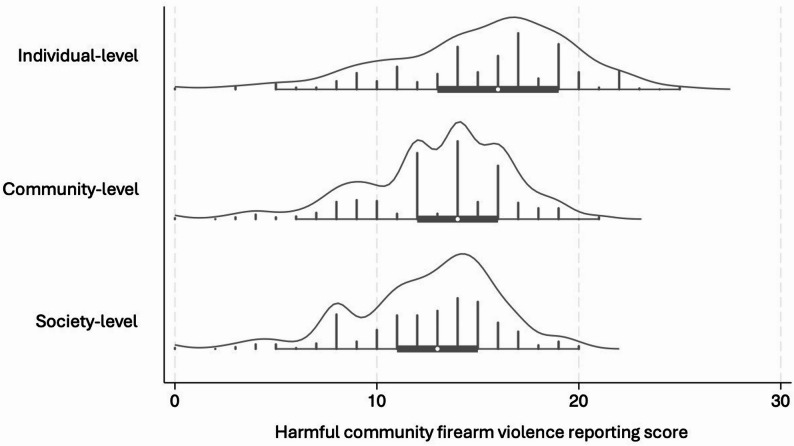
Distribution and kernel density plots of harmful community firearm violence scores at each socioecological level of harm. *Note.* Because of some differences in the potential severity ratings for each element across levels, the potential ranges are: 0–29 for individual-level harm, 0–25 for community-level harm, and 0–24 for society-level harm

**Fig. 3:**
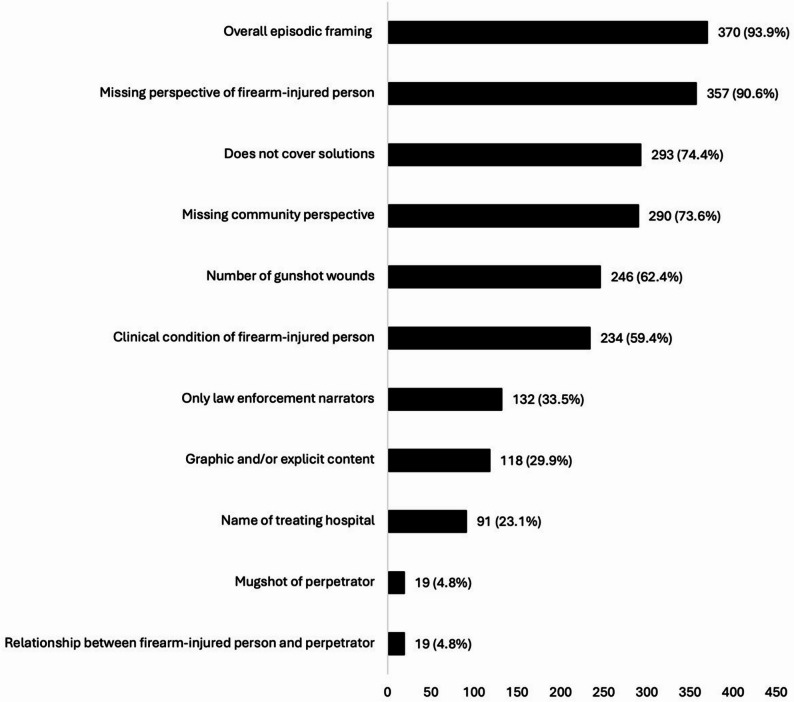
Frequency of each harmful community firearm violence reporting content elements among individuals (*N* = 394) covered in random selection of Philadelphia TV news clips, 2021

The median harmful CFV reporting scores for each level were 16 (IQR: 13–19, minimum: 0, maximum: 25) for the individual level, 14 (IQR: 12–16, minimum: 0, maximum: 21) for the community level, and 13 (IQR: 11–15, minimum: 0, maximum: 20) for the society level. Distributions of the scores at each level are depicted in Fig. 2. Harmful gun violence reporting elements from most to least common were overall episodic framing (93.8%), missing perspective of firearm-injured person (90.9%), does not cover solutions (73.3%), missing community perspective (72.6%), number of gunshot wounds (62.3%), clinical condition of firearm-injured person (59.3%), graphic and/or explicit content (30.3%), only law enforcement narrators (33.2%), name of treating hospital (22.4%), relationship between firearm-injured person and perpetrator (5.3%), and mugshot of perpetrator (4.8%) (Fig. 3). Pairwise correlations between the elements are provided in **Appendix D**.

### Generalized structural equation models

**Fig. 4:**
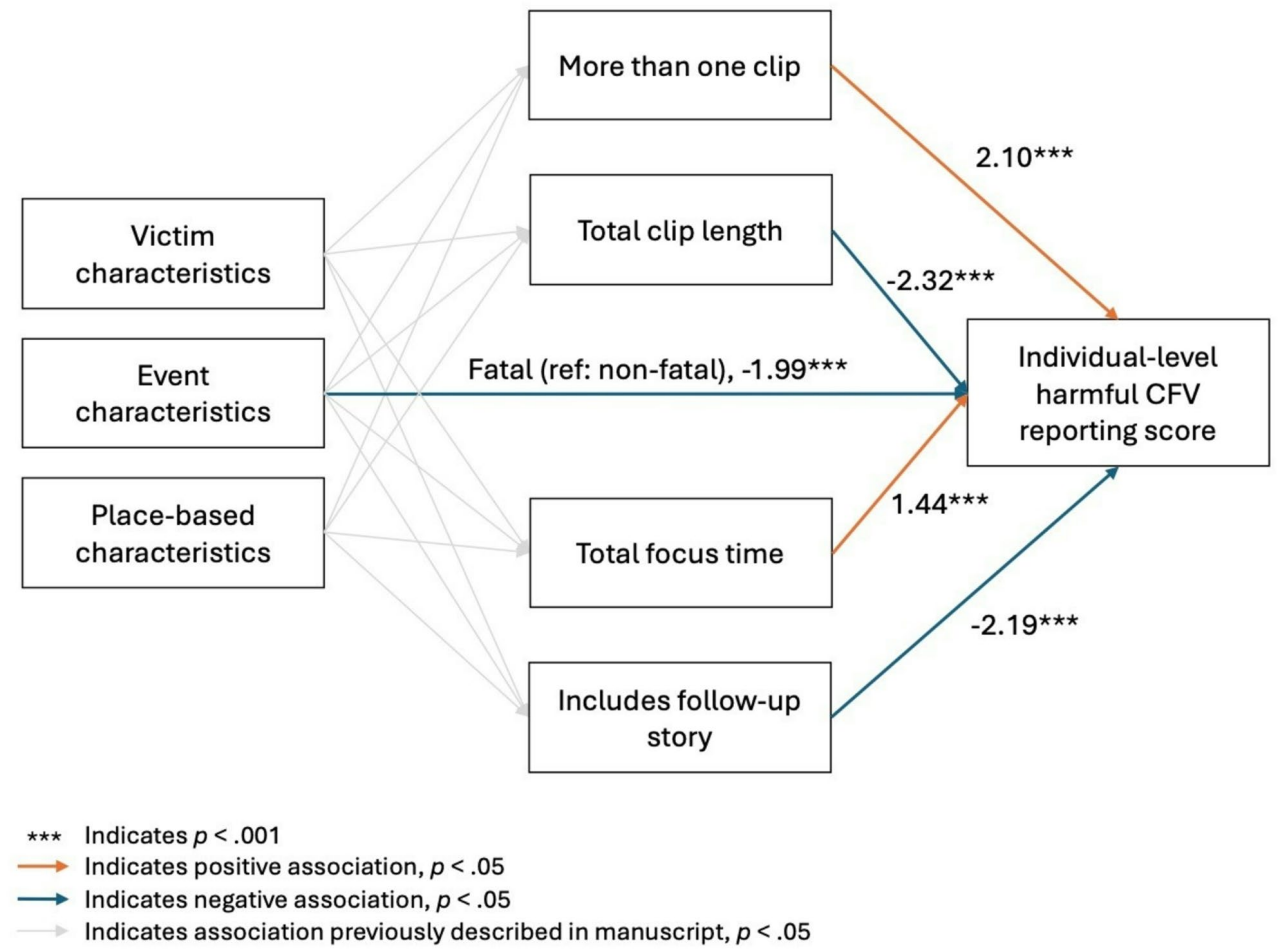
Direct paths to individual-level harmful community firearm violence reporting score

Figure 4 displays one of the 12 models we specified (i.e., for individual-level harmful gun violence reporting score). We did not specify models for the harmful reporting elements of “overall episodic framing,” and “missing perspective of firearm-injured person” due to frequencies > 0.90. For ease of interpretation, we report on only direction of association for all paths with *p* < .05.

#### Paths from victim, shooting event, and place-based characteristics to coverage characteristics

Because the same specification was used in all GSEMs, the associations between victim, shooting event, and place-based characteristics and coverage characteristics were the same across all models (Table 3). People under 18 (vs. people 18 and older) were more likely to have more than one clip and longer total clip length. Women (vs. men) were more likely to have longer total clip length, longer total focus time, and have coverage that includes a follow-up story. People shot in fatal events (vs. non-fatal) and mass shootings (vs. non-mass shooting) were more likely to have more than one clip, longer total clip length, longer total focus time, and have coverage that includes a follow-up story. People shot in places with a higher percentage of Black residents were less likely to have more than one clip, while those shot in places with a higher median household income were more likely to have more than one clip, have longer total focus time, and have coverage that includes a follow-up story. Income inequality was associated with all coverage characteristics, with greater income inequality being associated with a greater likelihood of having more than one clip, longer total clip length, longer total focus time, and having coverage that includes a follow-up story.

**Table 3:** Paths from victim, event, and place-based characteristics to coverage characteristics

Independent variable	Coverage characteristics							
	More than one clip (ref. one clip)		Total clip length		Total focus time		Includes follow-up story (ref. does not)	
	B	SE	B	SE	B	SE	B	SE
***Victim characteristics***								
Race/ethnicity								
Black	Ref.	-	Ref.	-	Ref.	-	Ref.	-
Hispanic	0.75	0.51	0.50	0.28	0.55	0.28	0.36	0.50
Asian	1.42	1.49	0.56	0.97	-0.44	0.94	-0.20	1.58
White	0.52	0.46	0.12	0.25	0.09	0.24	0.47	0.42
Age								
18 and older	Ref.	-	Ref.	-	Ref.	-	Ref.	-
Under 18	1.14***	**0.32**	0.40*	**0.18**	0.23	0.17	0.56	0.30
Sex								
Male	Ref.	-	Ref.	-	Ref.	-	Ref.	-
Female	0.27	0.34	0.45*	**0.19**	0.47*	**0.18**	0.80*	**0.31**
***Event characteristics***								
Fatal shooting								
Non-fatal	Ref.	-	Ref.	-	Ref.	-	Ref.	-
Fatal	1.20***	**0.29**	0.33*	**0.14**	0.36**	**0.14**	1.67***	**0.26**
Mass shooting								
Not a mass shooting	Ref.	-	Ref.	-	Ref.	-	Ref.	-
Mass shooting	2.21***	**0.40**	0.95***	**0.22**	0.97***	**0.21**	2.29***	**0.40**
***Place-based characteristics***								
Percent Black residents (CBG)	-0.32*	0.14	0.01	0.07	-0.06	0.07	-0.04	0.12
Percent of residents unemployed (CBG)	0.08	0.10	-0.07	0.05	-0.09	0.05	0.05	0.09
Median household income (CT)	0.46**	**0.18**	0.14	0.09	0.26**	**0.09**	0.51**	**0.16**
Percent poverty (CT)	-0.10	0.15	-0.07	0.08	0.00	0.08	-0.02	0.14
Income inequality (CT)	0.31**	**0.11**	0.18**	**0.06**	0.15**	**0.06**	0.32**	**0.10**
Racialized economic segregation (CT)	-0.29	0.19	0.08	0.10	-0.02	0.09	0.04	0.16

*Notes*. Unstandardized estimates from generalized structural equation models examining relationships from victim, event, place-based, and coverage characteristics to harmful gun violence reporting scale scores. Bolded values indicate *p* < .05

CBG = census block group; CT = census tract

* = *p* < .05; ** = *p* < .01; *** = *p* < .001

#### Direct paths from all characteristics to harmful CFV reporting scores

In the model with the dependent variable of individual-level harmful CFV reporting score, we saw direct paths to the outcome indicating that those shot in fatal shootings, with longer total clip length, and with coverage including a follow-up story had lower harmful CFV reporting score, while those having more than one clip and longer total focus time had higher harmful CFV reporting scores (Table 4). Directions were the same and associations were similar in magnitude for the models with the community-level and society-level harmful gun violence reporting score.

**Table 4:** Direct paths from victim, event, place-based, and coverage characteristics to harmful gun violence reporting scale scores

Independent variable	Harmful gun violence reporting scale scores by level					
	Individual		Community		Society	
	B	SE	B	SE	B	SE
***Victim characteristics***						
Race/ethnicity						
Black	Ref.	-	Ref.	-	Ref.	-
Hispanic	0.92	0.75	0.66	0.64	0.88	0.62
Asian	0.50	2.57	0.22	2.18	0.86	2.13
White	1.04	0.67	0.63	0.56	0.78	0.55
Age						
18 and older	Ref.	-	Ref.	-	Ref.	-
Under 18	-0.03	0.48	-0.24	0.40	-0.41	0.40
Sex						
Male	Ref.	-	Ref.	-	Ref.	-
Female	0.49	0.50	0/51	0.42	0.56	0.41
***Event characteristics***						
Fatal shooting						
Non-fatal	Ref.	-	Ref.	-	Ref.	-
Fatal	-1.99***	**0.40**	-1.72***	**0.34**	-1.25***	**0.33**
Mass shooting						
Not a mass shooting	Ref.	-	Ref.	-	Ref.	-
Mass shooting	-0.06	0.61	-0.22	0.51	-0.19	0.50
***Place-based characteristics***						
Percent Black residents (CBG)	-0.02	0.61	0.02	0.16	0.03	0.16
Percent of residents unemployed (CBG)	-0.22	0.13	-0.21	0.11	-0.15	0.11
Median household income (CT)	0.47	0.25	0.38	0.21	0.19	0.21
Percent poverty (CT)	0.24	0.21	0.18	0.18	0.11	0.18
Income inequality (CT)	-0.10	0.15	-0.07	0.13	-0.05	0.13
Racialized economic segregation (CT)	-0.34	0.25	-0.21	0.22	-0.06	0.21
***Coverage characteristics***						
Number of clips						
One clip	Ref.	-	Ref.	-	Ref.	-
More than one clip	2.10***	**0.57**	1.82***	**0.48**	1.62***	**0.47**
Total clip length	-2.32***	**0.30**	-1.93***	**0.25**	-1.91***	**0.24**
Total focus time	1.44***	**0.29**	1.36***	**0.25**	1.11***	**0.24**
Follow-up story						
Does not include follow-up story	Ref.	-	Ref.	-	Ref.	-
Includes follow-up story	-2.19***	**0.54**	-1.59***	**0.46**	-1.41**	**0.45**

*Notes*. Unstandardized estimates from generalized structural equation models examining relationships from victim, event, place-based, and coverage characteristics to harmful gun violence reporting scale scores. Bolded values indicate *p* < .05

CBG = census block group; CT = census tract

* = *p* < .05; ** = *p* < .01; *** = *p* < .001

#### Direct paths from all characteristics to harmful CFV reporting elements

Across models with the dependent variables of harmful gun violence reporting content elements, we saw the following direct paths to content elements (**Appendix E**). White people (vs. Black people) were less likely to have graphic and/or explicit content and more likely to have mugshot of perpetrator and only law enforcement narrators. People under 18 (vs. people 18 and older) were less likely to have graphic and/or explicit content and coverage that does not explore solutions, and more likely to have name of treating hospital. Women (vs. men) were more likely to have the relationship between firearm-injured person and perpetrator.

People shot in fatal shootings (vs. non-fatal) were less likely to have graphic and/or explicit content, clinical condition of firearm-injured person, and name of treating hospital. People shot in mass shootings (vs. non-mass shootings) were more likely to have clinical condition of firearm-injured person and coverage missing the community perspective, and less likely to have number of gunshot wounds.

People shot in places with a higher percentage of Black residents were more likely to have graphic and/or explicit content, and less likely to have clinical condition of firearm-injured person, missing the community perspective, and does not explore solutions. People shot in places with a higher percentage of residents who are unemployed were more likely to have missing community perspective, and less likely to have the relationship between firearm-injured person and perpetrator and does not cover solutions. People shot in communities with higher median household income were more likely to have name of treating hospital and does not explore solutions. People shot in areas with higher percentage of poverty were more likely to have name of treating hospital. People shot in places with higher income inequality were less likely to have mugshot of perpetrator. People shot in communities with a higher ICE score for racialized economic segregation (i.e., more concentrated privilege) were more likely to have graphic and/or explicit content, and less likely to have clinical condition of firearm-injured person, name of treating hospital, missing community perspective, and does not explore solutions.

Direct paths between coverage characteristics and each harmful CFV news content element, when statistically significant, generally resembled those for the total scores. The exception was that people whose coverage included a follow-up story (vs. did not) were more likely to have graphic and/or explicit content.

## Discussion

This study utilized a novel instrument and scale to quantify the frequency and severity of harmful CFV reporting on local TV news in Philadelphia. In addition to relating victim, shooting event, and place-based characteristics to harmful CFV reporting scores for the first time, we found that harmful CFV reporting was nearly universal. In this sample of TV news clips that mention a specific shooting victim in Philadelphia, just 6.1% of firearm-injured people were featured in a clip with thematic framing that included social context, epidemiological trends, root causes, or solutions. After overall episodic framing, the most common harmful CFV reporting content elements in TV news were missing perspective of firearm-injured person, does not explore solutions, missing community perspective, and number of gunshot wounds. Our analysis underscores the pervasive nature of harmful CFV news content, adds to our understanding of the public health burden of this harm, and urges journalists and news networks to re-examine reporting practices to limit harmful content.

Our use of GSEMs allowed us to tease out some of the complex associations between victim, shooting event, and place-based characteristics, news coverage-related characteristics, and harmful CFV reporting. Harmful CFV reporting scores and content elements in local TV news coverage appear to be shaped mostly indirectly by individual-level demographic, shooting event, and place-based characteristics, and more directly by differences in news coverage characteristics. Age, sex, and both shooting event characteristics had significant associations with coverage characteristics (e.g., children and women had longer total clip length), while all coverage characteristics in our analysis were significantly associated with harmful CFV reporting scores. Being involved in a fatal shooting was the only victim, shooting event, or place-based characteristic with a direct path to composite harmful CFV reporting scores, meaning people shot in fatal shootings had lower harmful CFV reporting scores regardless of their coverage characteristics. Together, these findings highlight how the choices journalists and news networks make to allocate resources to airtime and follow-up stories on specific types of shootings that occur in particular communities can disparately influence the extent of harm.

The disparities we uncovered in some specific harmful CFV content elements are especially urgent. In our previous research, Delphi panel experts identified graphic content as one of the most harmful CFV news content elements, with potential to cause severe harm at the individual, community, and society levels [25]. In the current study, we found that Black people, adult shooting victims, people shot in non-fatal shootings, and people shot in communities with a higher percentage of Black residents were more likely to have local TV news coverage that contains graphic and/or explicit content. News organizations generally refrain from publishing details about children who have been victims of crime [33], so it is unsurprising that stories about adult victims would include more disturbing content. It is cause for alarm that the likelihood of graphic and/or explicit content would be greater in the case of non-fatal shootings because survivors are vulnerable to being retraumatized by viewing news coverage of their shootings with such triggering material [31]. Additionally, these results are in line with previous research that has demonstrated that graphic imagery is more likely to be run in news coverage when the victims are non-white [48]. The disproportionate depiction of Black bodies ravaged by violence in the news fuels racist notions that Black deaths are unavoidable and inconsequential [19].

Assessing these relationships also highlighted important nuances to consider when quantifying harmful reporting. While we hypothesized that victim, shooting event, and place-based characteristics would be associated with harmful CFV TV news content, we also saw how the harm imposed by certain CFV content elements differed depending on the characteristics of the individual(s) shot. For example, white firearm-injured people (compared to Black firearm-injured people) were significantly more likely to be featured in clips with mugshots of the perpetrator, which counted toward to the harmful CFV reporting score for those white firearm-injured people in this study. To further explore this finding, we re-watched the clips which contained mugshots in our sample. Most mugshots featured Black men, a finding in line with communication literature that has repeatedly found that racially/ethnically minoritized people are overrepresented in crime story images and mugshots. Thus, in the case of mugshots in CFV on local TV news, the harms to society through the perpetuation of stereotypical narratives about Black people are likely more significant than harms to individual shooting victims featured in stories with mugshots. In response to these known harms, news outlets have reduced their utilization of mugshots in crime stories, recognizing their deleterious effects on individuals featured in the image and on society, especially in the digital era [49]. In addition, The Philadelphia Center for Gun Violence Reporting (PCGVR), a leading journalism ethics organization, recommends against using mugshots in CFV news stories as a best practice [50]. In future investigations of harmful CFV reporting, the harmful reporting score could be more aptly used to quantify society-level harm, while measures of each harmful content element can still allow for more granular examinations of multilevel disparities.

Some place-based characteristics appeared to shape both coverage characteristics and harmful CFV reporting, and the interpretation of these findings also requires close attention. People shot in places with higher median household income had more clips, longer total focus time, and more follow-up stories, while at the same time, people shot in communities with higher income inequality had more clips, longer total focus time, more follow-up stories, and longer total clip length. This could be because shootings in higher income areas are perceived as more newsworthy due to editorial priorities that place a premium on rarer, sensational shooting events, while in places with higher income inequality, contrasts between firearm-injured people and their surroundings may amplify perceived narrative value [21, 23]. We also saw how some content elements were more common in certain places in seemingly opposing ways. Graphic and/or explicit content was more common in coverage of individuals who were shot in census block groups with a greater percentage of Black residents, but also more common when individuals were shot in census tracts with higher ICE scores for racialized economic segregation, which indicates greater concentrated privilege (i.e., a greater proportion of high-income white people). This could reflect different reasons for news networks and journalists including graphic and/or explicit content, even if they both share the potential impact of sensationalizing CFV in these communities. An alternative explanation is that most CFV events occur in areas of Philadelphia with low median income and high social disadvantage and physical disorder [4, 51]. The locations where CFV events occur are largely homogeneous, meaning our sample had little variation in place-based characteristics.

Our findings can inform journalistic practice changes and shed light on opportunities to shape educational curricula for journalists on CFV reporting approaches that minimize harm. We found that the number of clips, total length, amount of focus, and decision of whether to have a follow-up story in the CFV news coverage of any given shooting is directly associated with the degree of harm. As such, stories that minimize focus time on a specific shooting victim or event in favor of exploration of root causes and solutions will be inherently less harmful than traditional episodic crime reports. In addition, including the content usually reserved for follow-up stories (which contained fewer harmful content elements in this study) instead of relying on frameworks for breaking CFV news coverage could be a valuable model for journalists. Our previous research has conceptualized the elements that constitute a public health frame in CFV reporting as: [1] epidemiologic context (through data or trends); [2] root causes; [3] public health narrators (e.g. public health professionals, community representatives, firearm-injured people and/or loved ones); [4] public health visuals (e.g. community events, interviews with community representatives, or firearm-injured people and/or loved ones); and [5] solutions (e.g. community violence intervention, investment in education, green spaces, social services) [14]. Educational efforts with journalists and in newsrooms can use these public health frame elements to shape news coverage templates for CFV stories to minimize harm.

This study should be interpreted with its limitations in mind. Our sample includes local TV news clips broadcast in a single city experiencing a surge in CFV in 2021. Therefore, our findings may not be generalizable to other cities in the US, to national TV news, or to print, radio, or social media content. In fact, it is possible that other television news stations across the US report firearm violence differently, which could have an impact on community perceptions and responses. At the same time, this study lays the methodological foundation future work to examine associations between harmful reporting scores and CFV incidence across news networks nationwide. Monitoring harmful CFV reporting with our novel harmful reporting score could be a way to track variation in harm over time and measure the impact of interventions aimed at minimizing harmful CFV news content. To this end, we focused on applying a summed score reflecting the level of harm that can facilitate comparison across clips rather than a scoring method that would indicate the presence or absence of certain elements or types of elements. Future analyses could consider examining other ways to group or score these elements. We also did not analytically account for repeated clips across more than one individual in our analyses; this could have created some imprecision in our GSEM estimates especially for less frequent elements such as mugshot of perpetrator and relationship between firearm-injured person and perpetrator. Future investigations could include analyses of associations between shooting circumstances, shooting location (e.g. public location vs. private residence) and harmful reporting scores to better inform our understanding of how other shooting characteristics relate to harmful reporting. Finally, because structural racism leads Black men to face a disproportionate burden of community firearm violence in many U.S. cities, our analysis was statistically limited in its ability to detect racial/ethnic and gender disparities because of relatively small numbers of individuals of some racial/ethnic groups and genders.

## Conclusions

In this study, we used a novel instrument and scale to quantify the frequency and severity of harmful CFV reporting on local TV news in Philadelphia. We hypothesized that victim, shooting event, and place-based characteristics influence both the general characteristics of CFV coverage and the inclusion of specific harmful content elements. Our results shed light on how news coverage characteristics can impact the extent of harm and provide insight into future measures of harmful CFV reporting. Perhaps most urgently, we uncovered that there are race- and place-based disparities in graphic and/or explicit content that need to be immediately addressed through the elimination of this type of content from CFV news reports. The results of this study can inform journalist education programs and guidance for journalistic practice to minimize harmful reporting and better inform the public on root causes and solutions to CFV.

## Supplementary Information

Below is the link to the electronic supplementary material.

Supplementary Material 1

Supplementary Material 2

Supplementary Material 3

Supplementary Material 4

Supplementary Material 5

## Data Availability

The linked dataset generated and used for all analyses in this study has been uploaded to the DSDR (Data Sharing for Demographic Research) of the ICPSR (Inter-university Consortium for Political and Social Research). The project ID is: DSDR-239263. The DOI is forthcoming. The dataset is also available from the authors on request.

## Abbreviations

CFV: Community firearm violence
GSEM: Generalized structural equation model
ICE: Index of Concentration at the Extremes
ICR: Intercoder reliability
PCGVR: Philadelphia Center for Gun Violence Reporting
PPD: Philadelphia Police Department
SD: Standard deviation
TV: Television
US: United States
