# Supplementary material for: Examining disparities in harmful reporting on community firearm violence in Philadelphia television news reports
Source: Inj Epidemiol. 2026 Feb 1;13:18. doi: 10.1186/s40621-026-00659-4 (PMC12952156; doi:10.1186/s40621-026-00659-4)
Supplement: Supplementary file 1 — Supplementary Material 1 [file 40621_2026_659_MOESM1_ESM.docx]

**Supplemental material for *Examining disparities in harmful reporting on community firearm violence in television news reports***

**Appendix A**. Harmful Reporting Codebook

| # | Variable / Field Name | Field Label  ***Field Note*** | Field Attributes (Field Type, Validation, Choices, Calculations, etc.) |
| --- | --- | --- | --- |
| Instrument: Harmful Reporting (harmful_reporting)  Enabled as survey | | | |
| 1 | [record_id] | Record ID | text |
| 2 | [coder_name] | Section Header: *Clip Information*  Name of Coder | text, Required |
| 3 | [clip_name] | What is the name of the clip? | text, Required |
| 4 | [clip_date] | On what date did the story air? | text (date_mdy), Required |
| 5 | [clip_newsstation] | What station produced the story? | \| 1 \| ABC \| \| --- \| --- \| \| 2 \| CBS \| \| 3 \| Fox \| \| 4 \| NBC \|   radio, Required |
| 6 | [clip_length] | What is the length of the entire clip, entered in minutes and seconds? (e.g.: 02:35) | text (time_mm_ss), Required |
| 7 | [shooting_location] | What location(s) does the story mention and/or show  ?  (Note ALL location information mentioned: neighborhood, block level, specific buildings, park, etc.) | notes, Required |
| 8 | [shooting_date] | On what date did the shooting occur? (Place date mentioned or deduced from the information provided. Otherwise select unknown and add information related to the date.)  *Write Unknown if not explicitly stated.* | \| 1 \| Exact Date {shooting_datespecific} \| \| --- \| --- \| \| 2 \| Exact Date Unknown  {shooting_dateapprox} \|   radio, Required |
| 9 | [shooting_datespecifi c]  Show the field ONLY if: [shooting_date]= '1' | Shooting Date | text |
| 10 | [shooting_dateapprox]  Show the field ONLY if: [shooting_date]= '2' | Approximate Shooting Date | text |
| 11 | [shooting_dateexplain] | What information did you use to get the date? | notes, Required |
| 12 | [shooting_time] | At what time did the shooting occur?  ( If no specific time is provided, note the general time of day, e.g., morning, afternoon, etc.) | \| 1 \| Exact time {shooting_timeexact} \| \| --- \| --- \| \| 2 \| Exact time Unknown  {shooting_timeapprox} \|   radio, Required |
| 13 | [shooting_timeexact]  Show the field ONLY if: [shooting_time] = '1' | Exact Shooting Time | text |

|  |  |
| --- | --- |
|  |  |

|  |  |
| --- | --- |
|  |  |
|  |  |
|  |  |
|  |  |
|  |  |
|  |  |
|  |  |
|  |  |
|  |  |
|  |  |

|  |  |
| --- | --- |
|  |  |
|  |  |

| 14 | [shooting_timeapprox]  Show the field ONLY if: [shooting_time] = '2' | Approximate Shooting time  *Any information related to the time of the shooting* | text |
| --- | --- | --- | --- |
| 15 | [shooting_episodic] | How much of the story is focused on a specific shooting event(s) ? (Enter in minutes: seconds) | text (time_mm_ss), Required |
| 16 | [shooting_thematic] | How much of the story is focused on firearm violence more broadly, including social context, epidemiological trends, statistics, root causes, solutions, impact on a community etc. ? (Enter in minutes: seconds) | text (time_mm_ss), Required |
| 17 | [shooting_framing] | What is the primary frame of this story? | radio, Required   1. Episodic (majority of time spent focused on specific shooting event(s)) 2. Thematic (majority of time spent discussing firearm violence more broadly, including social context, epidemiologic trends, root causes, solutions, impact on a community etc.) |
| 18 | [shooting_numvic] | Section Header: *Fill out for each victim*  How many shooting victims are mentioned? | dropdown, Required  1 1  2 2  3 3  4 4  5 5  6 6  7 7  8 8  9 9  10 10  11 11+ |
| 19 | [victim_whichone_1]  Show the field ONLY if: [shooting_numvic] = '1' o r [shooting_numvic] = '2' or [shooting_numvic] = '3' or [shooting_numvic]  = '3' or [shooting_numvi c] = '4' or [shooting_num vic] = '5' or [shooting_nu mvic] = '6' or [shooting_n umvic] = '7' or [shooting_ numvic] = '8' or [shooting  _numvic] = '9' or [shootin g_numvic] = '10' or [shoo ting_numvic] = '11' | Please note which victim you are coding for in the following responses. | text, Required |
| 20 | [victim_fatality] | Was the shooting fatal or non-fatal? | radio, Required   1. Fatal 2. Non-fatal 3. Unknown |

1. [victim_race]
2. [victim_raceeth_other]

Show the field ONLY if: [victim_race]= '7'

1. [victim_raceded]
2. [victim_raceother]

Show the field ONLY if: [victim_raceded(3)] = '1'

1. [victim_name]
2. [victim_namewritten]

Show the field ONLY if: [victim_name] = '2'

1. [victim_gender]
2. [victim_age]
3. [victim_agenum]

Show the field ONLY if: [victim_age] = '2'

1. [victim_additionalinf o]

What's the victim's race/ethnicity?

Other, please specify

How was race/ethnicity deduced? (Choose all that apply)

Other/Notes, please elaborate

Is the victim's name provided?

Victim's name

What is the victim's gender, if provided?

Is the victim's age provided?

Victim's age

Is any additional personal information provided about the victim?

radio, Required

| 1 | White |
| --- | --- |
| 2 | Latine |
| 3 | Black |
| 4 | Asian |
| 5 | Mixed Race/Multiracial |
| 6 | Unknown/Unclear from report |
| 7 | Other {victim_raceeth_other} |

text

checkbox, Required

| 1 | victim_raceded 1 | Explicitly mentioned verbally or textually in segment |
| --- | --- | --- |
| 2 | victim_raceded 2 | From a photograph or video visual |
| 3 | victim_raceded 3 | Other/Notes on thought process |
| 4 | victim_raceded 4 | N/A (no race/ethnicity information in clip) |

text

radio, Required

| 1 | No name provided |
| --- | --- |
| 2 | Name provided |

text

radio, Required

| 1 | Male |
| --- | --- |
| 2 | Female |
| 3 | Non-binary |
| 4 | Unknown/Unclear from report |

radio, Required

| 1 | No age provided |
| --- | --- |
| 2 | Age provided |

text

notes

|  |  | *Extra information such as hometown, accomplishments, community involvement, criminal record, parent, vocation,any adjective/descriptors, etc.* |  | | | |
| --- | --- | --- | --- | --- | --- | --- |
| 31 | [victim_shootingcircu m] | What are the circumstances of the shooting? (Can check multiple) | checkbox, Required | | | |
|  |  |  |  | 1 | victim_shootingcircum 1 | Interpersonal violence (including conflict, "gang- related", "drug- related", and/or "innocent bystander", etc.) |
|  |  |  |  | 2 | victim_shootingcircum 2 | Unintentional injury ("accidental" injury, e.g. firearm was not intentionally shot) |
|  |  |  |  | 3 | victim_shootingcircum 3 | Self-inflicted |
|  |  |  |  | 6 | victim_shootingcircum 6 | Officer- Involved Shooting (police shoots someone) |
|  |  |  |  | 7 | victim_shootingcircum 7 | Officer shot (someone shoots the officer) |
|  |  |  |  | 4 | victim_shootingcircum 4 | Unknown |
|  |  |  |  | 5 | victim_shootingcircum 5 | Other |
| 32 | [victim_circum_other]  Show the field ONLY if: [victim_shootingcircum (5)] = '1' | Other, please specify | notes | | | |
| 33 | [victim_whichone_2]  Show the field ONLY if: [shooting_numvic] = '2' o r [shooting_numvic] = '3' or [shooting_numvic] = '3' or [shooting_numvic]  = '4' or [shooting_numvi c] = '5' or [shooting_num vic] = '6' or [shooting_nu mvic] = '7' or [shooting_n umvic] = '8' or [shooting_ numvic] = '9' or [shooting  _numvic] = '10' or [shooti ng_numvic] = '11' | Victim#2:Please note which victim you are coding for in the following responses. | text, Required | | | |

| 1 | Fatal |
| --- | --- |
| 2 | Non-fatal |
| 3 | Unknown |

| 1 | White |
| --- | --- |
| 2 | Latine |
| 3 | Black |
| 4 | Asian |
| 5 | Mixed Race/Multiracial |
| 6 | Unknown/Unclear from report |
| 7 | Other |

| 1 | victim_raceded_2 1 | Explicitly mentioned verbally or textually in segment |
| --- | --- | --- |
| 2 | victim_raceded_2 2 | From a photograph or video visual |
| 3 | victim_raceded_2 3 | Other |
| 4 | victim_raceded_2 4 | N/A (no race/ethnicity information in clip) |

| 1 | No name provided |
| --- | --- |
| 2 | Name provided |

| 34 | [victim_fatality_2]  Show the field ONLY if: [shooting_numvic] = '2' o r [shooting_numvic] = '3' or [shooting_numvic] = '3' or [shooting_numvic]  = '4' or [shooting_numvi c] = '5' or [shooting_num vic] = '6' or [shooting_nu mvic] = '7' or [shooting_n umvic] = '8' or [shooting_ numvic] = '9' or [shooting  _numvic] = '10' or [shooti ng_numvic] = '11' | Victim #2: Was the shooting fatal or non-fatal? | radio, Required |
| --- | --- | --- | --- |
| 35 | [victim_race_2]  Show the field ONLY if: [shooting_numvic] = '2' o r [shooting_numvic] = '3' or [shooting_numvic] = '3' or [shooting_numvic]  = '4' or [shooting_numvi c] = '5' or [shooting_num vic] = '6' or [shooting_nu mvic] = '7' or [shooting_n umvic] = '8' or [shooting_ numvic] = '9' or [shooting  _numvic] = '10' or [shooti ng_numvic] = '11' | Victim #2: What's the victim's race/ethnicity? | radio, Required |
| 36 | [victim_raceeth_other_ 2]  Show the field ONLY if: [victim_race_2]= '7' | Other, please specify | text |
| 37 | [victim_raceded_2]  Show the field ONLY if: [shooting_numvic] = '2' o r [shooting_numvic] = '3' or [shooting_numvic] = '3' or [shooting_numvic]  = '4' or [shooting_numvi c] = '5' or [shooting_num vic] = '6' or [shooting_nu mvic] = '7' or [shooting_n umvic] = '8' or [shooting_ numvic] = '9' or [shooting  _numvic] = '10' or [shooti ng_numvic] = '11' | Victim #2: How was race/ethnicity deduced? | checkbox, Required |
| 38 | [victim_raceother_2]  Show the field ONLY if: [victim_raceded_2(3)] = '1' | Victim #2: Other, please specify | text |
| 39 | [victim_name_2]  Show the field ONLY if: [shooting_numvic] = '2' o r [shooting_numvic] = '3' | Victim #2: Is the victim's name provided? | radio, Required |

|  | or [shooting_numvic] = '3' or [shooting_numvic]  = '4' or [shooting_numvi c] = '5' or [shooting_num vic] = '6' or [shooting_nu mvic] = '7' or [shooting_n umvic] = '8' or [shooting_ numvic] = '9' or [shooting  _numvic] = '10' or [shooti ng_numvic] = '11' |  |  |
| --- | --- | --- | --- |
| 40 | [victim_namewriiten_2]  Show the field ONLY if: [victim_name_2] = '2' | Victim #2: Victim's name | text |
| 41 | [victim_gender_1]  Show the field ONLY if: [shooting_numvic] = '2' o r [shooting_numvic] = '3' or [shooting_numvic] = '3' or [shooting_numvic]  = '4' or [shooting_numvi c] = '5' or [shooting_num vic] = '6' or [shooting_nu mvic] = '7' or [shooting_n umvic] = '8' or [shooting_ numvic] = '9' or [shooting  _numvic] = '10' or [shooti ng_numvic] = '11' | Victim #2: What is the victim's gender, if provided? | radio, Required |
| 42 | [victim_age_2]  Show the field ONLY if: [shooting_numvic] = '2' o r [shooting_numvic] = '3' or [shooting_numvic] = '3' or [shooting_numvic]  = '4' or [shooting_numvi c] = '5' or [shooting_num vic] = '6' or [shooting_nu mvic] = '7' or [shooting_n umvic] = '8' or [shooting_ numvic] = '9' or [shooting  _numvic] = '10' or [shooti ng_numvic] = '11' | Victim #2: Is the victim's age provided? | radio, Required |
| 43 | [victim_agenum_2]  Show the field ONLY if: [victim_age_2] = '2' | Victim #2: Victim's age | text |
| 44 | [victim_additionalinfo  _1]  Show the field ONLY if: [shooting_numvic] = '2' o r [shooting_numvic] = '3' or [shooting_numvic] = '3' or [shooting_numvic]  = '4' or [shooting_numvi c] = '5' or [shooting_num vic] = '6' or [shooting_nu mvic] = '7' or [shooting_n umvic] = '8' or [shooting_  numvic] = '9' or [shooting | Victim #2: Is any additional personal information provided about the victim?  *Extra information such as hometown, accomplishments, community involvement, criminal record, any adjective/descriptors, etc.* | notes |

| 1 | Male |
| --- | --- |
| 2 | Female |
| 3 | Non-binary |
| 4 | Unknown/Unclear from report |

| 1 | No age provided |
| --- | --- |
| 2 | Age provided |

|  | _numvic] = '10' or [shooti ng_numvic] = '11' |  |  | | | |
| --- | --- | --- | --- | --- | --- | --- |
| 45 | [victim_shootingcircum  _2]  Show the field ONLY if: [shooting_numvic] = '2' o r [shooting_numvic] = '3' or [shooting_numvic] = '3' or [shooting_numvic]  = '4' or [shooting_numvi c] = '5' or [shooting_num vic] = '6' or [shooting_nu mvic] = '7' or [shooting_n umvic] = '8' or [shooting_ numvic] = '9' or [shooting  _numvic] = '10' or [shooti ng_numvic] = '11' | Victim #2: What are the circumstances of the shooting? (Can check multiple) | checkbox, Required | | | |
|  |  |  |  | 1 | victim_shootingcircum_2 1 | Interpersona violence (including conflict, "gang- related", "drug- related", and/or "innocent bystander", etc.) |
|  |  |  |  | 2 | victim_shootingcircum_2 2 | Unintentiona injury ("accidental" injury, e.g. firearm was not intentionally shot) |
|  |  |  |  | 3 | victim_shootingcircum_2 3 | Self-inflicted |
|  |  |  |  | 6 | victim_shootingcircum_2 6 | Officer- Involved Shooting (police shoots someone) |
|  |  |  |  | 7 | victim_shootingcircum_2 7 | Officer shot (someone shoots the officer) |
|  |  |  |  | 4 | victim_shootingcircum_2 4 | Unknown |
|  |  |  |  | 5 | victim_shootingcircum_2 5 | Other |
| 46 | [victim_circum_other_ 2]  Show the field ONLY if: [victim_shootingcircum_ 2(5)] = '1' | Other, please specify | notes | | | |
| 47 | [victim_whichone_3]  Show the field ONLY if: [shooting_numvic] = '3' o r [shooting_numvic] = '3' or [shooting_numvic] = '4' or [shooting_numvic]  = '5' or [shooting_numvi c] = '6' or [shooting_num vic] = '7' or [shooting_nu mvic] = '8' or [shooting_n umvic] = '9' or [shooting_ numvic] = '10' or [shooti ng_numvic] = '11' | Victim#3:Please note which victim you are coding for in the following responses. | text, Required | | | |
| 48 | [victim_fatality_3] | Victim #3: Was the shooting fatal or non-fatal? | radio, Required | | | |

|  | Show the field ONLY if: [shooting_numvic] = '3' o r [shooting_numvic] = '3' or [shooting_numvic] = '4' or [shooting_numvic]  = '5' or [shooting_numvi c] = '6' or [shooting_num vic] = '7' or [shooting_nu mvic] = '8' or [shooting_n umvic] = '9' or [shooting_ numvic] = '10' or [shooti ng_numvic] = '11' |  |  |
| --- | --- | --- | --- |
| 49 | [victim_race_3]  Show the field ONLY if: [shooting_numvic] = '3' o r [shooting_numvic] = '3' or [shooting_numvic] = '4' or [shooting_numvic]  = '5' or [shooting_numvi c] = '6' or [shooting_num vic] = '7' or [shooting_nu mvic] = '8' or [shooting_n umvic] = '9' or [shooting_ numvic] = '10' or [shooti ng_numvic] = '11' | Victim #3: What's the victim's race/ethnicity? | radio, Required |
| 50 | [victim_raceeth_other_ 3]  Show the field ONLY if: [victim_race_3]= '7' | Other, please specify | text |
| 51 | [victim_raceded_3]  Show the field ONLY if: [shooting_numvic] = '3' o r [shooting_numvic] = '3' or [shooting_numvic] = '4' or [shooting_numvic]  = '5' or [shooting_numvi c] = '6' or [shooting_num vic] = '7' or [shooting_nu mvic] = '8' or [shooting_n umvic] = '9' or [shooting_ numvic] = '10' or [shooti ng_numvic] = '11' | Victim #3: How was race/ethnicity deduced? | checkbox, Required |
| 52 | [victim_raceother_3]  Show the field ONLY if: [victim_raceded_3(3)] = '1' | Victim #3: Other, please specify | text |
| 53 | [victim_name_3]  Show the field ONLY if: [shooting_numvic] = '3' o r [shooting_numvic] = '3' or [shooting_numvic] = '4' or [shooting_numvic]  = '5' or [shooting_numvi c] = '6' or [shooting_num | Victim #3: Is the victim's name provided? | radio, Required |

| 1 | Fatal |
| --- | --- |
| 2 | Non-fatal |
| 3 | Unknown |

| 1 | White |
| --- | --- |
| 2 | Latine |
| 3 | Black |
| 4 | Asian |
| 5 | Mixed Race/Multiracial |
| 6 | Unknown/Unclear from report |
| 7 | Other |

| 1 | victim_raceded_3 1 | Explicitly mentioned verbally or textually in segment |
| --- | --- | --- |
| 2 | victim_raceded_3 2 | From a photograph or video visual |
| 3 | victim_raceded_3 3 | Other |
| 4 | victim_raceded_3 4 | N/A (no race/ethnicity information in clip) |

| 1 | No name provided |
| --- | --- |
| 2 | Name provided |

| 1 | Male |
| --- | --- |
| 2 | Female |
| 3 | Non-binary |
| 4 | Unknown/Unclear from report |

| 1 | No age provided |
| --- | --- |
| 2 | Age provided |

|  | vic] = '7' or [shooting_nu mvic] = '8' or [shooting_n umvic] = '9' or [shooting_ numvic] = '10' or [shooti ng_numvic] = '11' |  |  | | | |
| --- | --- | --- | --- | --- | --- | --- |
| 54 | [victim_namewriiten_3]  Show the field ONLY if: [victim_name_3] = '2' | Victim #3: Victim's name | text | | | |
| 55 | [victim_gender_3]  Show the field ONLY if: [shooting_numvic] = '3' o r [shooting_numvic] = '3' or [shooting_numvic] = '4' or [shooting_numvic]  = '5' or [shooting_numvi c] = '6' or [shooting_num vic] = '7' or [shooting_nu mvic] = '8' or [shooting_n umvic] = '9' or [shooting_ numvic] = '10' or [shooti ng_numvic] = '11' | Victim #3: What is the victim's gender, if provided? | radio, Required | | | |
| 56 | [victim_age_3]  Show the field ONLY if: [shooting_numvic] = '3' o r [shooting_numvic] = '3' or [shooting_numvic] = '4' or [shooting_numvic]  = '5' or [shooting_numvi c] = '6' or [shooting_num vic] = '7' or [shooting_nu mvic] = '8' or [shooting_n umvic] = '9' or [shooting_ numvic] = '10' or [shooti ng_numvic] = '11' | Victim #3: Is the victim's age provided? | radio, Required | | | |
| 57 | [victim_agenum_3]  Show the field ONLY if: [victim_age_3] = '2' | Victim #3: Victim's age | text | | | |
| 58 | [victim_additionalinfo  _3]  Show the field ONLY if: [shooting_numvic] = '3' o r [shooting_numvic] = '3' or [shooting_numvic] = '4' or [shooting_numvic]  = '5' or [shooting_numvi c] = '6' or [shooting_num vic] = '7' or [shooting_nu mvic] = '8' or [shooting_n umvic] = '9' or [shooting_ numvic] = '10' or [shooti ng_numvic] = '11' | Victim #3: Is any additional personal information provided about the victim?  *Extra information such as hometown, accomplishments, community involvement, criminal record, any adjective/descriptors, etc.* | notes | | | |
| 59 | [victim_shootingcircum  _3]  Show the field ONLY if: [shooting_numvic] = '3' o r [shooting_numvic] = '3' | Victim #3: What are the circumstances of the shooting? (Can choose multiple) | checkbox, Required | | | |
|  |  |  |  | 1 | victim_shootingcircum_3 1 | Interpersona violence (including conflict, |

|  | or [shooting_numvic] = '4' or [shooting_numvic]  = '5' or [shooting_numvi c] = '6' or [shooting_num vic] = '7' or [shooting_nu mvic] = '8' or [shooting_n umvic] = '9' or [shooting_ numvic] = '10' or [shooti ng_numvic] = '11' |  |  |  |  | "gang- related", "drug- related", and/or "innocent bystander", etc.) |
| --- | --- | --- | --- | --- | --- | --- |
|  |  |  |  | 2 | victim_shootingcircum_3 2 | Unintentiona injury ("accidental" injury, e.g. firearm was not intentionally shot) |
|  |  |  |  | 3 | victim_shootingcircum_3 3 | Self-inflicted |
|  |  |  |  | 6 | victim_shootingcircum_3 6 | Officer- Involved Shooting (police shoots someone) |
|  |  |  |  | 7 | victim_shootingcircum_3 7 | Officer shot (someone shoots the officer) |
|  |  |  |  | 4 | victim_shootingcircum_3 4 | Unknown |
|  |  |  |  | 5 | victim_shootingcircum_3 5 | Other |
| 60 | [victim_circum_other_ 3]  Show the field ONLY if: [victim_shootingcircum_ 3(5)] = '1' | Other, please specify | notes | | | |
| 61 | [victim_whichone_4]  Show the field ONLY if: [shooting_numvic] = '4' o r [shooting_numvic] = '5' or [shooting_numvic] = '6' or [shooting_numvic]  = '7' or [shooting_numvi c] = '8' or [shooting_num vic] = '9' or [shooting_nu mvic] = '10' or [shooting_ numvic] = '11' | Victim#4: Please note which victim you are coding for in the following responses. | text, Required | | | |
| 62 | [victim_fatality_4]  Show the field ONLY if: [shooting_numvic] = '4' o r [shooting_numvic] = '5' or [shooting_numvic] = '6' or [shooting_numvic]  = '7' or [shooting_numvi c] = '8' or [shooting_num vic] = '9' or [shooting_nu | Victim #4: Was the shooting fatal or non-fatal? | radio, Required   1. Fatal 2. Non-fatal 3. Unknown | | | |

|  |  |
| --- | --- |
|  |  |
|  |  |

|  | mvic] = '10' or [shooting_ numvic] = '11' |  |  |
| --- | --- | --- | --- |
| 63 | [victim_race_4]  Show the field ONLY if: [shooting_numvic] = '4' o r [shooting_numvic] = '5' or [shooting_numvic] = '6' or [shooting_numvic]  = '7' or [shooting_numvi c] = '8' or [shooting_num vic] = '9' or [shooting_nu mvic] = '10' or [shooting_ numvic] = '11' | Victim #4: What's the victim's race/ethnicity? | radio, Required |
| 64 | [victim_raceeth_other_ 4]  Show the field ONLY if: [victim_race_4]= '7' | Other, please specify | text |
| 65 | [victim_raceded_4]  Show the field ONLY if: [shooting_numvic] = '4' o r [shooting_numvic] = '5' or [shooting_numvic] = '6' or [shooting_numvic]  = '7' or [shooting_numvi c] = '8' or [shooting_num vic] = '9' or [shooting_nu mvic] = '10' or [shooting_ numvic] = '11' | Victim #4: How was race/ethnicity deduced? | checkbox, Required |
| 66 | [victim_raceother_4]  Show the field ONLY if: [victim_raceded_4(3)] = '1' | Victim #4: Other, please specify | text |
| 67 | [victim_name_4]  Show the field ONLY if: [shooting_numvic] = '4' o r [shooting_numvic] = '5' or [shooting_numvic] = '6' or [shooting_numvic]  = '7' or [shooting_numvi c] = '8' or [shooting_num vic] = '9' or [shooting_nu mvic] = '10' or [shooting_ numvic] = '11' | Victim #4: Is the victim's name provided? | radio, Required |
| 68 | [victim_namewriiten_4]  Show the field ONLY if: [victim_name_4] = '2' | Victim #4: Victim's name | text |
| 69 | [victim_gender_4]  Show the field ONLY if: [shooting_numvic] = '4' o r [shooting_numvic] = '5' | Victim #4: What is the victim's gender, if provided? | radio, Required |

| 1 | White |
| --- | --- |
| 2 | Latine |
| 3 | Black |
| 4 | Asian |
| 5 | Mixed Race/Multiracial |
| 6 | Unknown/Unclear from report |
| 7 | Other |

| 1 | victim_raceded_4 1 | Explicitly mentioned verbally or textually in segment |
| --- | --- | --- |
| 2 | victim_raceded_4 2 | From a photograph or video visual |
| 3 | victim_raceded_4 3 | Other |
| 4 | victim_raceded_4 4 | N/A (no race/ethnicity information in clip) |

| 1 | No name provided |
| --- | --- |
| 2 | Name provided |

| 1 | Male |
| --- | --- |
| 2 | Female |

| 3 | Non-binary |
| --- | --- |
| 4 | Unknown/Unclear from report |

| 1 | No age provided |
| --- | --- |
| 2 | Age provided |

|  | or [shooting_numvic] = '6' or [shooting_numvic]  = '7' or [shooting_numvi c] = '8' or [shooting_num vic] = '9' or [shooting_nu mvic] = '10' or [shooting_ numvic] = '11' |  |  | | | |
| --- | --- | --- | --- | --- | --- | --- |
| 70 | [victim_age_4]  Show the field ONLY if: [shooting_numvic] = '4' o r [shooting_numvic] = '5' or [shooting_numvic] = '6' or [shooting_numvic]  = '7' or [shooting_numvi c] = '8' or [shooting_num vic] = '9' or [shooting_nu mvic] = '10' or [shooting_ numvic] = '11' | Victim #4: Is the victim's age provided? | radio, Required | | | |
| 71 | [victim_agenum_4]  Show the field ONLY if: [victim_age_4] = '2' | Victim #4: Victim's age | text | | | |
| 72 | [victim_additionalinfo  _4]  Show the field ONLY if: [shooting_numvic] = '4' o r [shooting_numvic] = '5' or [shooting_numvic] = '6' or [shooting_numvic]  = '7' or [shooting_numvi c] = '8' or [shooting_num vic] = '9' or [shooting_nu mvic] = '10' or [shooting_ numvic] = '11' | Victim #4: Is any additional personal information provided about the victim?  *Extra information such as hometown, accomplishments, community involvement, criminal record, any adjective/descriptors, etc.* | notes | | | |
| 73 | [victim_shootingcircum  _4]  Show the field ONLY if: [shooting_numvic] = '4' o r [shooting_numvic] = '5' or [shooting_numvic] = '6' or [shooting_numvic]  = '7' or [shooting_numvi c] = '8' or [shooting_num vic] = '9' or [shooting_nu mvic] = '10' or [shooting_ numvic] = '11' | Victim #4: What are the circumstances of the shooting? (Can choose multiple) | checkbox, Required | | | |
|  |  |  |  | 1 | victim_shootingcircum_4 1 | Interpersona violence (including conflict, "gang- related", "drug- related", and/or "innocent bystander", etc.) |
|  |  |  |  | 2 | victim_shootingcircum_4 2 | Unintentiona injury ("accidental" injury, e.g. firearm was not intentionally shot) |
|  |  |  |  | 3 | victim_shootingcircum_4 3 | Self-inflicted |
|  |  |  |  | | | |

|  |  |  |  | 6 | victim_shootingcircum_4 6 | Officer- Involved Shooting (police shoots someone) |
| --- | --- | --- | --- | --- | --- | --- |
|  |  |  |  | 7 | victim_shootingcircum_4 7 | Officer shot (someone shoots the officer) |
|  |  |  |  | 4 | victim_shootingcircum_4 4 | Unknown |
|  |  |  |  | 5 | victim_shootingcircum_4 5 | Other |
| 74 | [victim_circum_other_ 4]  Show the field ONLY if: [victim_shootingcircum_ 4(5)] = '1' | Other, please specify | notes | | | |
| 75 | [victim_whichone_5]  Show the field ONLY if: [shooting_numvic] = '5' o r [shooting_numvic] = '6' or [shooting_numvic] = '7' or [shooting_numvic]  = '8' or [shooting_numvi c] = '9' or [shooting_num vic] = '10' or [shooting_n umvic] = '11' | Victim#5: Please note which victim you are coding for in the following responses. | text, Required | | | |
| 76 | [victim_fatality_5]  Show the field ONLY if: [shooting_numvic] = '5' o r [shooting_numvic] = '6' or [shooting_numvic] = '7' or [shooting_numvic]  = '8' or [shooting_numvi c] = '9' or [shooting_num vic] = '10' or [shooting_n umvic] = '11' | Victim #5: Was the shooting fatal or non-fatal? | radio, Required   1. Fatal 2. Non-fatal 3. Unknown | | | |
| 77 | [victim_race_5]  Show the field ONLY if: [shooting_numvic] = '5' o r [shooting_numvic] = '6' or [shooting_numvic] = '7' or [shooting_numvic]  = '8' or [shooting_numvi c] = '9' or [shooting_num vic] = '10' or [shooting_n umvic] = '11' | Victim #5: What's the victim's race/ethnicity? | radio, Required   1. White 2. Latine 3. Black 4. Asian 5. Mixed Race/Multiracial 6. Unknown/Unclear from report 7. Other | | | |
| 78 | [victim_raceeth_other_ 5]  Show the field ONLY if: [victim_race_5]= '7' | Other, please specify | text | | | |
| 79 | [victim_raceded_5] | Victim #5: How was race/ethnicity deduced? | checkbox, Required | | | |

|  |  |
| --- | --- |
|  |  |
|  |  |

|  |  |
| --- | --- |
|  |  |
|  |  |
|  |  |
|  |  |
|  |  |
|  |  |

|  | Show the field ONLY if: [shooting_numvic] = '5' o r [shooting_numvic] = '6' or [shooting_numvic] = '7' or [shooting_numvic]  = '8' or [shooting_numvi c] = '9' or [shooting_num vic] = '10' or [shooting_n umvic] = '11' |  |  |
| --- | --- | --- | --- |
| 80 | [victim_raceother_5]  Show the field ONLY if: [victim_raceded_5(3)] = '1' | Victim #5: Other, please specify | text |
| 81 | [victim_name_5]  Show the field ONLY if: [shooting_numvic] = '5' o r [shooting_numvic] = '6' or [shooting_numvic] = '7' or [shooting_numvic]  = '8' or [shooting_numvi c] = '9' or [shooting_num vic] = '10' or [shooting_n umvic] = '11' | Victim #5: Is the victim's age provided? | radio, Required   1. No name provided 2. Name provided |
| 82 | [victim_namewriiten_5]  Show the field ONLY if: [victim_name_5] = '2' | Victim #5: Victim's name | text |
| 83 | [victim_gender_5]  Show the field ONLY if: [shooting_numvic] = '5' o r [shooting_numvic] = '6' or [shooting_numvic] = '7' or [shooting_numvic]  = '8' or [shooting_numvi c] = '9' or [shooting_num vic] = '10' or [shooting_n umvic] = '11' | Victim #5: What is the victim's gender, if provided? | radio, Required   1. Male 2. Female 3. Non-binary 4. Unknown/Unclear from report |
| 84 | [victim_age_5]  Show the field ONLY if: [shooting_numvic] = '5' o r [shooting_numvic] = '6' or [shooting_numvic] = '7' or [shooting_numvic]  = '8' or [shooting_numvi c] = '9' or [shooting_num vic] = '10' or [shooting_n umvic] = '11' | Victim #5: Is the victim's age provided? | radio, Required   1. No age provided 2. Age provided |
| 85 | [victim_agenum_5]  Show the field ONLY if: [victim_age_5] = '2' | Victim #5: Victim's age | text |

| 1 | victim_raceded_5 1 | Explicitly mentioned verbally or textually in segment |
| --- | --- | --- |
| 2 | victim_raceded_5 2 | From a photograph or video visual |
| 3 | victim_raceded_5 3 | Other |
| 4 | victim_raceded_5 4 | N/A (no race/ethnicity information in clip) |

|  |  |
| --- | --- |
|  |  |

|  |  |
| --- | --- |
|  |  |
|  |  |
|  |  |

|  |  |
| --- | --- |
|  |  |

| 86 | [victim_additionalinfo  _5]  Show the field ONLY if: [shooting_numvic] = '5' o r [shooting_numvic] = '6' or [shooting_numvic] = '7' or [shooting_numvic]  = '8' or [shooting_numvi c] = '9' or [shooting_num vic] = '10' or [shooting_n umvic] = '11' | Victim #5: Is any additional personal information provided about the victim?  *Extra information such as hometown, accomplishments, community involvement, criminal record, any adjective/descriptors, etc.* | notes | | | |
| --- | --- | --- | --- | --- | --- | --- |
| 87 | [victim_shootingcircum  _5]  Show the field ONLY if: [shooting_numvic] = '5' o r [shooting_numvic] = '6' or [shooting_numvic] = '7' or [shooting_numvic]  = '8' or [shooting_numvi c] = '9' or [shooting_num vic] = '10' or [shooting_n umvic] = '11' | Victim #5: What are the circumstances of the shooting? (Can choose multiple) | checkbox, Required | | | |
|  |  |  |  | 1 | victim_shootingcircum_5 1 | Interpersona violence (including conflict, "gang- related", "drug- related", and/or "innocent bystander", etc.) |
|  |  |  |  | 2 | victim_shootingcircum_5 2 | Unintentiona injury ("accidental" injury, e.g. firearm was not intentionally shot) |
|  |  |  |  | 3 | victim_shootingcircum_5 3 | Self-inflicted |
|  |  |  |  | 6 | victim_shootingcircum_5 6 | Officer- Involved Shooting (police shoots someone) |
|  |  |  |  | 7 | victim_shootingcircum_5 7 | Officer shot (someone shoots the officer) |
|  |  |  |  | 4 | victim_shootingcircum_5 4 | Unknown |
|  |  |  |  | 5 | victim_shootingcircum_5 5 | Other |
| 88 | [victim_circum_other_ 5]  Show the field ONLY if: [victim_shootingcircum_ 5(5)] = '1' | Other, please specify | notes | | | |
| 89 | [victim_whichone_6]  Show the field ONLY if: [shooting_numvic] = '6' o r [shooting_numvic] = '7' or [shooting_numvic] = | Victim#6: Please note which victim you are coding for in the following responses. | text, Required | | | |

| 1 | Fatal |
| --- | --- |
| 2 | Non-fatal |
| 3 | Unknown |

| 1 | White |
| --- | --- |
| 2 | Latine |
| 3 | Black |
| 4 | Asian |
| 5 | Mixed Race/Multiracial |
| 6 | Unknown/Unclear from report |
| 7 | Other |

| 1 | victim_raceded_6 1 | Explicitly mentioned verbally or textually in segment |
| --- | --- | --- |
| 2 | victim_raceded_6 2 | From a photograph or video visual |
| 3 | victim_raceded_6 3 | Other |
| 4 | victim_raceded_6 4 | N/A (no race/ethnicity information in clip) |

| 1 | No name provided |
| --- | --- |
| 2 | Name provided |

|  | '8' or [shooting_numvic]  = '9' or [shooting_numvi c] = '10' or [shooting_nu mvic] = '11' |  |  |
| --- | --- | --- | --- |
| 90 | [victim_fatality_6]  Show the field ONLY if: [shooting_numvic] = '6' o r [shooting_numvic] = '7' or [shooting_numvic] = '8' or [shooting_numvic]  = '9' or [shooting_numvi c] = '10' or [shooting_nu mvic] = '11' | Victim #6: Was the shooting fatal or non-fatal? | radio, Required |
| 91 | [victim_race_6]  Show the field ONLY if: [shooting_numvic] = '6' o r [shooting_numvic] = '7' or [shooting_numvic] = '8' or [shooting_numvic]  = '9' or [shooting_numvi c] = '10' or [shooting_nu mvic] = '11' | Victim #6: What's the victim's race/ethnicity? | radio, Required |
| 92 | [victim_raceeth_other_ 6]  Show the field ONLY if: [victim_race_6]= '7' | Other, please specify | text |
| 93 | [victim_raceded_6]  Show the field ONLY if: [shooting_numvic] = '6' o r [shooting_numvic] = '7' or [shooting_numvic] = '8' or [shooting_numvic]  = '9' or [shooting_numvi c] = '10' or [shooting_nu mvic] = '11' | Victim #6: How was race/ethnicity deduced? | checkbox, Required |
| 94 | [victim_raceother_6]  Show the field ONLY if: [victim_raceded_6(3)] = '1' | Victim #6: Other, please specify | text |
| 95 | [victim_name_6]  Show the field ONLY if: [shooting_numvic] = '6' o r [shooting_numvic] = '7' or [shooting_numvic] = '8' or [shooting_numvic]  = '9' or [shooting_numvi | Victim #6: Is the victim's name provided? | radio, Required |

|  | c] = '10' or [shooting_nu mvic] = '11' |  | | | |
| --- | --- | --- | --- | --- | --- |
| 96 | [victim_namewriiten_6] | Victim #6: Victim's name |  | text |  |
|  | Show the field ONLY if: [victim_name_6] = '2' |  |  |  |  |
| 97 | [victim_gender_6] | Victim #6: What is the victim's gender, if provided? |  | radio, Required |  |
|  | Show the field ONLY if: |  |  |  |  |
|  | [shooting_numvic] = '6' o r [shooting_numvic] = '7'  or [shooting_numvic] = |  |  |  |  |
|  | '8' or [shooting_numvic]  = '9' or [shooting_numvi c] = '10' or [shooting_nu mvic] = '11' |  |  |  |  |
| 98 | [victim_age_6] | Victim #6: Is the victim's age provided? |  | radio, Required |  |
|  | Show the field ONLY if: |  |  |  |  |
|  | [shooting_numvic] = '6' o r [shooting_numvic] = '7' or [shooting_numvic] = '8' or [shooting_numvic]  = '9' or [shooting_numvi c] = '10' or [shooting_nu mvic] = '11' |  |  |  |  |
| 99 | [victim_agenum_6] | Victim #6: Victim's age |  | text |  |
|  | Show the field ONLY if: [victim_age_6] = '2' |  |  |  |  |
| 100 | [victim_additionalinfo  _6] | Victim #6: Is any additional personal information provided about the victim? |  | notes |  |

| 1 | Male |
| --- | --- |
| 2 | Female |
| 3 | Non-binary |
| 4 | Unknown/Unclear from report |

| 1 | No age provided |
| --- | --- |
| 2 | Age provided |

101

Show the field ONLY if: [shooting_numvic] = '6' o r [shooting_numvic] = '7' or [shooting_numvic] = '8' or [shooting_numvic]

= '9' or [shooting_numvi c] = '10' or [shooting_nu mvic] = '11'

[victim_shootingcircum

_6]

Show the field ONLY if: [shooting_numvic] = '6' o r [shooting_numvic] = '7' or [shooting_numvic] = '8' or [shooting_numvic]

= '9' or [shooting_numvi c] = '10' or [shooting_nu mvic] = '11'

*Extra information such as hometown, accomplishments, community involvement, criminal record, any adjective/descriptors, etc.*

Victim #6: What are the circumstances of the shooting? (Can choose multiple)

checkbox, Required

1. victim_shootingcircum_6 1
2. victim_shootingcircum_6 2

Interpersona violence (including conflict, "gang- related", "drug- related", and/or "innocent bystander", etc.)

Unintentiona injury ("accidental" injury, e.g. firearm was not

|  |  |
| --- | --- |
|  |  |
|  |  |

|  |  |
| --- | --- |
|  |  |
|  |  |
|  |  |
|  |  |
|  |  |
|  |  |

|  |  |  |  |  |  | intentionally shot) |
| --- | --- | --- | --- | --- | --- | --- |
|  |  |  |  | 3 | victim_shootingcircum_6 3 | Self-inflicted |
|  |  |  |  | 6 | victim_shootingcircum_6 6 | Officer- Involved Shooting (police shoots someone) |
|  |  |  |  | 7 | victim_shootingcircum_6 7 | Officer shot (someone shoots the officer) |
|  |  |  |  | 4 | victim_shootingcircum_6 4 | Unknown |
|  |  |  |  | 5 | victim_shootingcircum_6 5 | Other |
| 102 | [victim_circum_other_ 6]  Show the field ONLY if: [victim_shootingcircum_ 6(5)] = '1' | Other, please specify | notes | | | |
| 103 | [victim_whichone_7]  Show the field ONLY if: [shooting_numvic] = '7' o r [shooting_numvic] = '8' or [shooting_numvic] = '9' or [shooting_numvic]  = '10' or [shooting_numvi c] = '11' | Victim#7: Please note which victim you are coding for in the following responses. | text, Required | | | |
| 104 | [victim_fatality_7]  Show the field ONLY if: [shooting_numvic] = '7' o r [shooting_numvic] = '8' or [shooting_numvic] = '9' or [shooting_numvic]  = '10' or [shooting_numvi c] = '11' | Victim #7: Was the shooting fatal or non-fatal? | radio, Required   1. Fatal 2. Non-fatal 3. Unknown | | | |
| 105 | [victim_race_7]  Show the field ONLY if: [shooting_numvic] = '7' o r [shooting_numvic] = '8' or [shooting_numvic] = '9' or [shooting_numvic]  = '10' or [shooting_numvi c] = '11' | Victim #7: What's the victim's race/ethnicity? | radio, Required   1. White 2. Latine 3. Black 4. Asian 5. Mixed Race/Multiracial 6. Unknown/Unclear from report 7. Other | | | |
| 106 | [victim_raceeth_other_ 7]  Show the field ONLY if: [victim_race_7]= '7' | Other, please specify | text | | | |
| 107 | [victim_raceded_7]  Show the field ONLY if: | Victim #7: How was race/ethnicity deduced? | checkbox, Required | | | |

[shooting_numvic] = '7' o r [shooting_numvic] = '8' or [shooting_numvic] = '9' or [shooting_numvic]

| 1 | victim_raceded_7 1 | Explicitly mentioned verbally or textually in segment |
| --- | --- | --- |
| 2 | victim_raceded_7 2 | From a photograph or video visual |
| 3 | victim_raceded_7 3 | Other |
| 4 | victim_raceded_7 4 | N/A (no race/ethnicity information in clip) |

= '10' or [shooting_numvi c] = '11'

108

109

110

111

112

113

114

[victim_raceother_7]

Show the field ONLY if: [victim_raceded_7(3)] = '1'

[victim_name_7]

Show the field ONLY if: [shooting_numvic] = '7' o r [shooting_numvic] = '8' or [shooting_numvic] = '9' or [shooting_numvic]

= '10' or [shooting_numvi c] = '11'

[victim_namewriiten_7]

Show the field ONLY if: [victim_name_7] = '2'

[victim_gender_7]

Show the field ONLY if: [shooting_numvic] = '7' o r [shooting_numvic] = '8' or [shooting_numvic] = '9' or [shooting_numvic]

= '10' or [shooting_numvi c] = '11'

[victim_age_7]

Show the field ONLY if: [shooting_numvic] = '7' o r [shooting_numvic] = '8' or [shooting_numvic] = '9' or [shooting_numvic]

= '10' or [shooting_numvi c] = '11'

[victim_agenum_7]

Show the field ONLY if: [victim_age_7] = '2'

[victim_additionalinfo

_7]

Show the field ONLY if: [shooting_numvic] = '7' o r [shooting_numvic] = '8'

Victim #7: Other, please specify

Victim #7: Is the victim's name provided?

Victim #7: Victim's name

Victim #7: What is the victim's gender, if provided?

Victim #7: Is the victim's age provided?

Victim #7: Victim's age

Victim #7: Is any additional personal information provided about the victim?

*Extra information such as hometown, accomplishments, community involvement, criminal record, any adjective/descriptors, etc.*

text

radio, Required

| 1 | No name provided |
| --- | --- |
| 2 | Name provided |

text

radio, Required

| 1 | Male |
| --- | --- |
| 2 | Female |
| 3 | Non-binary |
| 4 | Unknown/Unclear from report |

radio, Required

| 1 | No age provided |
| --- | --- |
| 2 | Age provided |

text

notes

|  | or [shooting_numvic] = '9' or [shooting_numvic]  = '10' or [shooting_numvi c] = '11' |  |  | | | |
| --- | --- | --- | --- | --- | --- | --- |
| 115 | [victim_shootingcircum  _7]  Show the field ONLY if: [shooting_numvic] = '7' o r [shooting_numvic] = '8' or [shooting_numvic] = '9' or [shooting_numvic]  = '10' or [shooting_numvi c] = '11' | Victim #7: What are the circumstances of the shooting? (Can choose multiple) | checkbox, Required | | | |
|  |  |  |  | 1 | victim_shootingcircum_7 1 | Interpersona violence (including conflict, "gang- related", "drug- related", and/or "innocent bystander", etc.) |
|  |  |  |  | 2 | victim_shootingcircum_7 2 | Unintentiona injury ("accidental" injury, e.g. firearm was not intentionally shot) |
|  |  |  |  | 3 | victim_shootingcircum_7 3 | Self-inflicted |
|  |  |  |  | 6 | victim_shootingcircum_7 6 | Officer- Involved Shooting (police shoots someone) |
|  |  |  |  | 7 | victim_shootingcircum_7 7 | Officer shot (someone shoots the officer) |
|  |  |  |  | 4 | victim_shootingcircum_7 4 | Unknown |
|  |  |  |  | 5 | victim_shootingcircum_7 5 | Other |
| 116 | [victim_circum_other_ 7]  Show the field ONLY if: [victim_shootingcircum_ 7(5)] = '1' | Other, please specify | notes | | | |
| 117 | [victim_whichone_8]  Show the field ONLY if: [shooting_numvic] = '8' o r [shooting_numvic] = '9' or [shooting_numvic] = '1 0' or [shooting_numvic] = '11' | Victim #8: Please note which victim you are coding for in the following responses. | text, Required | | | |

118

119

120

121

122

123

124

125

[victim_fatality_8]

Show the field ONLY if: [shooting_numvic] = '8' o r [shooting_numvic] = '9' or [shooting_numvic] = '1 0' or [shooting_numvic] = '11'

[victim_race_8]

Show the field ONLY if: [shooting_numvic] = '8' o r [shooting_numvic] = '9' or [shooting_numvic] = '1 0' or [shooting_numvic] = '11'

[victim_raceeth_other_ 8]

Show the field ONLY if: [victim_race_8]= '7'

[victim_raceded_8]

Show the field ONLY if: [shooting_numvic] = '8' o r [shooting_numvic] = '9' or [shooting_numvic] = '1 0' or [shooting_numvic] = '11'

[victim_raceother_8]

Show the field ONLY if: [victim_raceded_8(3)] = '1'

[victim_name_8]

Show the field ONLY if: [shooting_numvic] = '8' o r [shooting_numvic] = '9' or [shooting_numvic] = '1 0' or [shooting_numvic] = '11'

[victim_namewriiten_8]

Show the field ONLY if: [victim_name_8] = '2'

[victim_gender_8]

Show the field ONLY if:

Victim #8: Was the shooting fatal or non-fatal?

Victim #8: What's the victim's race/ethnicity?

Other, please specify

Victim #8: How was race/ethnicity deduced?

Victim #8: Other, please specify

Victim #8: Is the victim's name provided?

Victim #8: Victim's name

Victim #8: What is the victim's gender, if provided?

radio, Required

| 1 | Fatal |
| --- | --- |
| 2 | Non-fatal |
| 3 | Unknown |

radio, Required

| 1 | White |
| --- | --- |
| 2 | Latine |
| 3 | Black |
| 4 | Asian |
| 5 | Mixed Race/Multiracial |
| 6 | Unknown/Unclear from report |
| 7 | Other |

text

checkbox, Required

| 1 | victim_raceded_8 1 | Explicitly mentioned verbally or textually in segment |
| --- | --- | --- |
| 2 | victim_raceded_8 2 | From a photograph or video visual |
| 3 | victim_raceded_8 3 | Other |
| 4 | victim_raceded_8 4 | N/A (no race/ethnicity information in clip) |

text

radio, Required

| 1 | No name provided |
| --- | --- |
| 2 | Name provided |

text

radio, Required

| 1 | Male |
| --- | --- |

| 2 | Female |
| --- | --- |
| 3 | Non-binary |
| 4 | Unknown/Unclear from report |

| 1 | No age provided |
| --- | --- |
| 2 | Age provided |

|  | [shooting_numvic] = '8' o r [shooting_numvic] = '9' or [shooting_numvic] = '1 0' or [shooting_numvic] = '11' |  |  | | | |
| --- | --- | --- | --- | --- | --- | --- |
| 126 | [victim_age_8]  Show the field ONLY if: [shooting_numvic] = '8' o r [shooting_numvic] = '9' or [shooting_numvic] = '1 0' or [shooting_numvic] = '11' | Victim #8: Is the victim's age provided? | radio, Required | | | |
| 127 | [victim_agenum_8]  Show the field ONLY if: [victim_age_8] = '2' | Victim #8: Victim's age | text | | | |
| 128 | [victim_additionalinfo  _8]  Show the field ONLY if: [shooting_numvic] = '8' o r [shooting_numvic] = '9' or [shooting_numvic] = '1 0' or [shooting_numvic] = '11' | Victim #8: Is any additional personal information provided about the victim?  *Extra information such as hometown, accomplishments, community involvement, criminal record, any adjective/descriptors, etc.* | notes | | | |
| 129 | [victim_shootingcircum | Victim #8: What are the circumstances of the shooting? (Can choose multiple) | checkbox, Required | | | |
|  | _8]  Show the field ONLY if: [shooting_numvic] = '8' o r [shooting_numvic] = '9' or [shooting_numvic] = '1 0' or [shooting_numvic] = '11' |  |  | 1 | victim_shootingcircum_8 1 | Interpersona violence (including conflict, "gang- related", "drug- related", and/or "innocent bystander", etc.) |
|  |  |  |  | 2 | victim_shootingcircum_8 2 | Unintentiona injury ("accidental" injury, e.g. firearm was not intentionally shot) |
|  |  |  |  | 3 | victim_shootingcircum_8 3 | Self-inflicted |
|  |  |  |  | 6 | victim_shootingcircum_8 6 | Officer- Involved Shooting (police shoots someone) |
|  |  |  |  | 7 | victim_shootingcircum_8 7 | Officer shot (someone shoots the officer) |
|  |  |  |  | | | |

|  |  |
| --- | --- |
|  |  |
|  |  |

|  |  |
| --- | --- |
|  |  |
|  |  |
|  |  |
|  |  |
|  |  |
|  |  |

|  |  |  |
| --- | --- | --- |
|  |  |  |
|  |  |  |
|  |  |  |

|  |  |  |  | 4 | victim_shootingcircum_8 4 | Unknown |
| --- | --- | --- | --- | --- | --- | --- |
|  |  |  |  | 5 | victim_shootingcircum_8 5 | Other |
| 130 | [victim_circum_other_ 8]  Show the field ONLY if: [victim_shootingcircum_ 8(5)] = '1' | Other, please specify | notes | | | |
| 131 | [victim_whichone_9]  Show the field ONLY if: [shooting_numvic] = '9' o r [shooting_numvic] = '1 0' or [shooting_numvic] = '11' | Victim#9: Please note which victim you are coding for in the following responses. | text, Required | | | |
| 132 | [victim_fatality_9]  Show the field ONLY if: [shooting_numvic] = '9' o r [shooting_numvic] = '1 0' or [shooting_numvic] = '11' | Victim #9: Was the shooting fatal or non-fatal? | radio, Required   1. Fatal 2. Non-fatal 3. Unknown | | | |
| 133 | [victim_race_9]  Show the field ONLY if: [shooting_numvic] = '9' o r [shooting_numvic] = '1 0' or [shooting_numvic] = '11' | Victim #9: What's the victim's race/ethnicity? | radio, Required   1. White 2. Latine 3. Black 4. Asian 5. Mixed Race/Multiracial 6. Unknown/Unclear from report 7. Other | | | |
| 134 | [victim_raceeth_other_ 9]  Show the field ONLY if: [victim_race_9]= '7' | Other, please specify | text | | | |
| 135 | [victim_raceded_9]  Show the field ONLY if: [shooting_numvic] = '9' o r [shooting_numvic] = '1 0' or [shooting_numvic] = '11' | Victim #9: How was race/ethnicity deduced? | checkbox, Required   1. victim_raceded_9 1 Explicitly   mentioned verbally or textually in segment   1. victim_raceded_9 2 From a   photograph or video visual   1. victim_raceded_9 3 Other 2. victim_raceded_9 4 N/A (no   race/ethnicity information in clip) | | | |
| 136 | [victim_raceother_9]  Show the field ONLY if: [victim_raceded_9(3)] = '1' | Victim #9: Other, please specify | text | | | |

137

138

139

140

141

142

143

[victim_name_9]

Show the field ONLY if: [shooting_numvic] = '9' o r [shooting_numvic] = '1 0' or [shooting_numvic] = '11'

[victim_namewriiten_9]

Show the field ONLY if: [victim_name_9] = '2'

[victim_gender_9]

Show the field ONLY if: [shooting_numvic] = '9' o r [shooting_numvic] = '1 0' or [shooting_numvic] = '11'

[victim_age_9]

Show the field ONLY if: [shooting_numvic] = '9' o r [shooting_numvic] = '1 0' or [shooting_numvic] = '11'

[victim_agenum_9]

Show the field ONLY if: [victim_age_9] = '2'

[victim_additionalinfo

_9]

Show the field ONLY if: [shooting_numvic] = '9' o r [shooting_numvic] = '1 0' or [shooting_numvic] = '11'

[victim_shootingcircum

Victim #9: Is the victim's name provided?

Victim #9: Victim's name

Victim #9: What is the victim's gender, if provided?

Victim #9: Is the victim's age provided?

Victim #9: Victim's age

Victim #9: Is any additional personal information provided about the victim?

*Extra information such as hometown, accomplishments, community involvement, criminal record, any adjective/descriptors, etc.*

Victim #9: What are the circumstances of the

radio, Required

| 1 | No name provided |
| --- | --- |
| 2 | Name provided |

text

radio, Required

| 1 | Male |
| --- | --- |
| 2 | Female |
| 3 | Non-binary |
| 4 | Unknown/Unclear from report |

radio, Required

| 1 | No age provided |
| --- | --- |
| 2 | Age provided |

text

notes

checkbox, Required

_9]

Show the field ONLY if: [shooting_numvic] = '9' o r [shooting_numvic] = '1 0' or [shooting_numvic] = '11'

shooting? (Can choose multiple)

1. victim_shootingcircum_9 1
2. victim_shootingcircum_9 2
3. victim_shootingcircum_9 3

Interpersona violence (including conflict, "gang- related", "drug- related", and/or "innocent bystander", etc.)

Unintentiona injury ("accidental" injury, e.g. firearm was not intentionally shot)

Self-inflicted

|  |  |  |  | 6 | victim_shootingcircum_9 6 | Officer- Involved Shooting (police shoots someone) |
| --- | --- | --- | --- | --- | --- | --- |
|  |  |  |  | 7 | victim_shootingcircum_9 7 | Officer shot (someone shoots the officer) |
|  |  |  |  | 4 | victim_shootingcircum_9 4 | Unknown |
|  |  |  |  | 5 | victim_shootingcircum_9 5 | Other |
| 144 | [victim_circum_other_ 9]  Show the field ONLY if: [victim_shootingcircum_ 9(5)] = '1' | Other, please specify | notes | | | |
| 145 | [victim_whichone_10]  Show the field ONLY if: [shooting_numvic] = '10' or [shooting_numvic] = '1 1' | Victim #10: Please note which victim you are coding for in the following responses. | text, Required | | | |
| 146 | [victim_fatality_10]  Show the field ONLY if: [shooting_numvic] = '10' or [shooting_numvic] = '1 1' | Victim #10: Was the shooting fatal or non-fatal? | radio, Required | | | |
| 147 | [victim_race_10]  Show the field ONLY if: [shooting_numvic] = '10' or [shooting_numvic] = '1 1' | Victim #10: What's the victim's race/ethnicity? | radio, Required | | | |
| 148 | [victim_raceeth_other_ 10]  Show the field ONLY if: [victim_race_10]= '7' | Other, please specify | text | | | |
| 149 | [victim_raceded_10]  Show the field ONLY if: [shooting_numvic] = '10' or [shooting_numvic] = '1 1' | Victim #10: How was race/ethnicity deduced? | checkbox, Required | | | |

| 1 | Fatal |
| --- | --- |
| 2 | Non-fatal |
| 3 | Unknown |

| 1 | White |
| --- | --- |
| 2 | Latine |
| 3 | Black |
| 4 | Asian |
| 5 | Mixed Race/Multiracial |
| 6 | Unknown/Unclear from report |
| 7 | Other |

| 1 | victim_raceded_10 1 | Explicitly mentioned verbally or textually in segment |
| --- | --- | --- |
| 2 | victim_raceded_10 2 | From a photograph or video visual |
| 3 | victim_raceded_10 3 | Other |

| 4 | victim_raceded_10 4 | N/A (no race/ethnicity information in clip) |
| --- | --- | --- |

150

151

152

153

154

155

156

157

[victim_raceother_10]

Show the field ONLY if: [victim_raceded_10(3)] = '1'

[victim_name_10]

Show the field ONLY if: [shooting_numvic] = '10' or [shooting_numvic] = '1 1'

[victim_namewriiten_1 0]

Show the field ONLY if: [victim_name_10] = '2'

[victim_gender_10]

Show the field ONLY if: [shooting_numvic] = '10' or [shooting_numvic] = '1 1'

[victim_age_10]

Show the field ONLY if: [shooting_numvic] = '10' or [shooting_numvic] = '1 1'

[victim_agenum_10]

Show the field ONLY if: [victim_age_10] = '2'

[victim_additionalinfo

_10]

Show the field ONLY if: [shooting_numvic] = '10' or [shooting_numvic] = '1 1'

[victim_shootingcircum

_10]

Show the field ONLY if: [shooting_numvic] = '10' or [shooting_numvic] = '1 1'

Victim #10: Other, please specify

Victim #10: Is the victim's name provided?

Victim #10: Victim's name

Victim #10: What is the victim's gender, if provided?

Victim #10: Is the victim's age provided?

Victim #10: Victim's age

Victim #10: Is any additional personal information provided about the victim?

*Extra information such as hometown, accomplishments, community involvement, criminal record, any adjective/descriptors, etc.*

Victim #10: What are the circumstances of the shooting?

text

radio, Required

| 1 | No name provided |
| --- | --- |
| 2 | Name provided |

text

radio, Required

| 1 | Male |
| --- | --- |
| 2 | Female |
| 3 | Non-binary |
| 4 | Unknown/Unclear from report |

radio, Required

| 1 | No age provided |
| --- | --- |
| 2 | Age provided |

text

notes

checkbox, Required

1. victim_shootingcircum_10 1
2. victim_shootingcircum_10 2

Interperson violence (including conflict, "gang- related", "drug- related", and/or "innocent bystander", etc.)

Unintention injury

"

|  |  |  |  |  |  | ("accidental injury, e.g. firearm was not intentionall shot) |
| --- | --- | --- | --- | --- | --- | --- |
|  |  |  |  | 3 | victim_shootingcircum_10 3 | Self-inflicted |
|  |  |  |  | 6 | victim_shootingcircum_10 6 | Officer- Involved Shooting (police shoots someone) |
|  |  |  |  | 7 | victim_shootingcircum_10 7 | Officer shot (someone shoots the officer) |
|  |  |  |  | 4 | victim_shootingcircum_10 4 | Unknown |
|  |  |  |  | 5 | victim_shootingcircum_10 5 | Other |
| 158 | [victim_circum_other_1 0]  Show the field ONLY if: [victim_shootingcircum_ 10(5)] = '1' | Other, please specify | notes | | | |
| 159 | [victim_whichone_11]  Show the field ONLY if: [shooting_numvic] = '11' | Victim #11: Please note which victim you are coding for in the following responses. | text, Required | | | |
| 160 | [victim_fatality_11]  Show the field ONLY if: [shooting_numvic] = '11' | Victim #11: Was the shooting fatal or non-fatal? | radio, Required | | | |
| 161 | [victim_race_11]  Show the field ONLY if: [shooting_numvic] = '11' | Victim #11: What's the victim's race/ethnicity? | radio, Required | | | |
| 162 | [victim_raceeth_other_ 11]  Show the field ONLY if: [victim_race_11]= '7' | Other, please specify | text | | | |
| 163 | [victim_raceded_11]  Show the field ONLY if: [shooting_numvic] = '11' | Victim #11: How was race/ethnicity deduced? | checkbox, Required  1 victim_raceded_11 1 Explicitly  mentioned verbally or textually in segment | | | |

y

| 1 | Fatal |
| --- | --- |
| 2 | Non-fatal |
| 3 | Unknown |

| 1 | White |
| --- | --- |
| 2 | Latine |
| 3 | Black |
| 4 | Asian |
| 5 | Mixed Race/Multiracial |
| 6 | Unknown/Unclear from report |
| 7 | Other |

|  |  |  |
| --- | --- | --- |

| 2 | victim_raceded_11 2 | From a photograph or video visual |
| --- | --- | --- |
| 3 | victim_raceded_11 3 | Other |
| 4 | victim_raceded_11 4 | N/A (no race/ethnicity information in clip) |

164

165

166

167

168

169

170

171

[victim_raceother_11]

Show the field ONLY if: [victim_raceded_11(3)] = '1' and [shooting_numvi

c] = '11'

[victim_name_11]

Show the field ONLY if: [shooting_numvic] = '11'

[victim_namewriiten_1 1]

Show the field ONLY if: [victim_name_11] = '2'

[victim_gender_11]

Show the field ONLY if: [shooting_numvic] = '11'

[victim_age_11]

Show the field ONLY if: [shooting_numvic] = '11'

[victim_agenum_11]

Show the field ONLY if: [victim_age_11] = '2'

[victim_additionalinfo

_11]

Show the field ONLY if: [shooting_numvic] = '11'

[victim_shootingcircum

_11]

Show the field ONLY if: [shooting_numvic] = '11'

Victim #11: Other, please specify

Victim #11: Is the victim's name provided?

Victim #11: Victim's name

Victim #11: What is the victim's gender, if provided?

Victim #11: Is the victim's age provided?

Victim #11: Victim's age

Victim #11: Is any additional personal information provided about the victim?

*Extra information such as hometown, accomplishments, community involvement, criminal record, any adjective/descriptors, etc.*

Victim #11: What are the circumstances of the shooting? (Can choose multiple)

text

radio, Required

| 1 | No name provided |
| --- | --- |
| 2 | Name provided |

text

radio, Required

| 1 | Male |
| --- | --- |
| 2 | Female |
| 3 | Non-binary |
| 4 | Unknown/Unclear from report |

radio, Required

| 1 | No age provided |
| --- | --- |
| 2 | Age provided |

text

notes

checkbox, Required

1 victim_shootingcircum_11 1

Interperson violence (including conflict, "gang- related", "drug- related", and/or "innocent bystander", etc.)

|  |  |  |  | 2 | victim_shootingcircum_11 2 | Unintention injury ("accidental injury, e.g. firearm was not intentionall shot) |
| --- | --- | --- | --- | --- | --- | --- |
|  |  |  |  | 3 | victim_shootingcircum_11 3 | Self-inflicted |
|  |  |  |  | 6 | victim_shootingcircum_11 6 | Officer- Involved Shooting (police shoots someone) |
|  |  |  |  | 7 | victim_shootingcircum_11 7 | Officer shot (someone shoots the officer) |
|  |  |  |  | 4 | victim_shootingcircum_11 4 | Unknown |
|  |  |  |  | 5 | victim_shootingcircum_11 5 | Other |
| 172 | [victim_circum_other_1 1]  Show the field ONLY if: [victim_shootingcircum_ 11(5)] = '1' | Other, please specify | notes | | | |
| 173 | [harm_condition] | Does the story include information on the clinical condition of the shooting victim beyond indicating if it is fatal or non-fatal (e.g. critical vs. stable, in the ICU, fighting for their life, in surgery, expected to recover, will survive)? | yesno, Required  1 Yes  0 No | | | |
| 174 | [harm_gsw] | Does the story include information about the gunshot wounds sustained by a shooting victim (including actual number and/or single, several, multiple, riddled, location)? | yesno, Required  1 Yes  0 No | | | |
| 175 | [harm_hosp] | Does the story include the name of the treating hospital of the shooting victim? | yesno, Required  1 Yes  0 No | | | |
| 176 | [harm_relation] | Does the story include information about the relationship between the shooting victim and the alleged perpetrator? | radio, Required | | | |
| 177 | [harm_solution] | Section Header: *Harmful Content Information*  Does the story include any mention of specific solutions for community firearm violence? | yesno, Required  1 Yes  0 No | | | |
| 178 | [harm_solution_yes]  Show the field ONLY if: [harm_solution] = '1' | Please specify which solutions are mentioned and/or discussed. | notes | | | |

"

y

|  |  |
| --- | --- |
|  |  |

| 1 | Yes (including statement that there no relationship) |
| --- | --- |
| 0 | No (including a statement that the relationship is unknown) |

| 1 | Yes |
| --- | --- |
| 0 | No |
| 2 | Unknown |

| 179 | [harm_perpetrator] | Does the story include a mugshot(s) or a police- related headshot of the alleged perpetrator(s)? | yesno, Required  1 Yes  0 No | | | |
| --- | --- | --- | --- | --- | --- | --- |
| 180 | [harm_followup] | Is the story a follow-up? A follow-up story is defined as one after the initial "breaking news" coverage (e.g. an update on how a community fared after a shooting, or an interview with a survivor about their recovery, etc.). | radio, Required | | | |
| 181 | [harm_graphiccontent] | Does the story include graphic and/or explicit content about firearm violence (e.g. video of a shooting, still photo of a body, audio of screaming, etc.)? | yesno, Required  1 Yes  0 No | | | |
| 182 | [harm_graphiccontentsp ecify]  Show the field ONLY if: [harm_graphiccontent] = '1' | Please specify the graphic and/or explicit content present in the story? | notes | | | |
| 183 | [harm_policeimagery] | Is police imagery present (e.g. police car, siren, police on scene, shell casings with numbers next to it, crime tape, including "B-roll")? | yesno  1 Yes  0 No | | | |
| 184 | [harm_journalist] | Is anyone besides journalist featured in including an "A-roll" interview (video or audio), a quotation, a paraphrased specific comment and/or a text quote graphic? | yesno, Required  1 Yes  0 No | | | |
| 185 | [harm_policesay] | Is any version of "police say" present in the story (including information attributed to police, "authorities", law enforcement, "police think", etc. ) ? | yesno, Required  1 Yes  0 No | | | |
| 186 | [harm_lovedonesintervi ew]  Show the field ONLY if: [harm_journalist] = '1' | Does the story include the perspective of the shooting victim and/or their loved ones, or people who knew the victim personally? This would include an "A-roll" interview (video or audio), a quotation, and/or text quote graphic. | yesno  1 Yes  0 No | | | |
| 187 | [harm_community]  Show the field ONLY if: [harm_journalist] = '1' | Does the story include the perspective of people from the impacted community (e.g. people impacted by previous shootings, community leaders, gun violence prevention advocates). This would include an "A-roll" interview (video or audio), a quotation, a paraphrased specific comment, and/or text quote graphic." | yesno  1 Yes  0 No | | | |
| 188 | [harm_leoperspective]  Show the field ONLY if: [harm_journalist] = '1' | Does the story include the perspective of law enforcement (e.g. any police representative, any district attorney representative)? This would include an "A-roll" interview, a quotation, a paraphrased specific comment, and/or a text quote graphic. | yesno  1 Yes  0 No | | | |
| 189 | [harm_speakerintervie w]  Show the field ONLY if: [harm_journalist] = '1' | Who is interviewed, shown speaking, heard speaking, and/or quoted directly in the story aside from journalists? Check all that apply and include total time (in minutes and seconds) that each narrator is shown speaking, heard speaking, and/or quoted directly in the video. | checkbox | | | |
|  |  |  |  | 1 | harm_speakerinterview 1 | Law Enforcement (Police and/or District Attorney)  {harm_leoint} |
|  |  |  |  | | | |

|  |  |  |  | 2 | harm_speakerinterview 2 | Shooting victi and/or loved ones (includin family, self- described clos friends, and spokesperson  {harm_vicint} |
| --- | --- | --- | --- | --- | --- | --- |
|  |  |  |  | 3 | harm_speakerinterview 3 | People from impacted community (including neighbors, witnesses, community leaders)  {harm_comint |
|  |  |  |  | 4 | harm_speakerinterview 4 | Other  {harm_otherin |
| 190 | [harm_leoint]  Show the field ONLY if: [harm_speakerinterview (1)] = '1' | Please list the person(s) and the time they are speaking in (MM:SS) | notes | | | |
| 191 | [harm_vicint]  Show the field ONLY if: [harm_speakerinterview (2)] = '1' | Please list the person(s) and the time they are speaking in (MM:SS) | notes | | | |
| 192 | [harm_comint]  Show the field ONLY if: [harm_speakerinterview (3)] = '1' | Please list the person(s) and the time they are speaking in (MM:SS) | notes | | | |
| 193 | [harm_otherint]  Show the field ONLY if: [harm_speakerinterview (4)] = '1' | Please specify other, and time spoken or quoted in (MM:SS) format. | notes | | | |
| 194 | [harm_leoprimaryinterv iew] | Is the primary narrator law enforcement (e.g. a law enforcement representative is shown speaking, heard speaking, and/or quoted directly in the story longer than any other narrator)? | yesno, Required  1 Yes  0 No | | | |
| 195 | [notes] | Please add any comments that might be helpful about this clip and how you coded it. For example, if you were torn on an answer, if there was a technical issue with the clip, or if any thoughts about the study in general came up, note those thoughts here. | notes | | | |
| 196 | [harmful_reporting_com plete] | Section Header: *Form Status*  Complete? | dropdown | | | |

)

| 0 | Incomplete |
| --- | --- |
| 1 | Unverified |
| 2 | Complete |
