# Supplementary material for: Examining disparities in harmful reporting on community firearm violence in Philadelphia television news reports
Source: Inj Epidemiol. 2026 Feb 1;13:18. doi: 10.1186/s40621-026-00659-4 (PMC12952156; doi:10.1186/s40621-026-00659-4)
Supplement: Supplementary file 2 — Supplementary Material 2 [file 40621_2026_659_MOESM2_ESM.docx]

**Supplemental material for *Examining disparities in harmful reporting on community firearm violence in television news reports***

**Appendix B.** Intercoder Reliability Scores for Quantitative Media Content Analysis

| **Variable** | **Gwet's AC_1_** | **Krippendorff's Alpha** | **Percent Agreement** |
| --- | --- | --- | --- |
| Exact Date Included | 0.811 | 0.743 | 0.891 |
| Exact Time Included | 0.932 | 0.930 | 0.966 |
| Number of Victims | 0.946 | 0.901 | 0.954 |
| Name Included | 0.980 | 0.969 | 0.988 |
| Sex Included | 0.967 | 0.954 | 0.976 |
| Race Included | 0.968 | 0.896 | 0.976 |
| Age Included | 0.938 | 0.911 | 0.963 |
| Fatal or Non-fatal | 0.910 | 0.863 | 0.932 |
| Graphic Content Included | 0.922 | 0.783 | 0.943 |
| Clinical Condition Included | 0.886 | 0.860 | 0.937 |
| Number of Gunshots Included | 0.875 | 0.873 | 0.937 |
| Hospital Name Included | 1.0 | 1.0 | 1.0 |
| Relationship Included | 0.974 | 0.878 | 0.978 |
| Mugshot Included | 0.976 | 0.938 | 0.983 |
| Episodic Framing | 0.935 | 0.742 | 0.948 |
| Law Enforcement Narrator | 1.0 | 1.0 | 1.0 |
| “Police Say” Included | 0.863 | 0.722 | 0.908 |
| Non-police Interviewed | 0.970 | 0.959 | 0.983 |
| Police Imagery Included | 0.979 | 0.900 | 0.983 |
| Solutions Included | 0.936 | 0.840 | 0.954 |
| Follow-up Story | 0.786 | 0.753 | 0.885 |
