# Supplementary material for: Examining disparities in harmful reporting on community firearm violence in Philadelphia television news reports
Source: Inj Epidemiol. 2026 Feb 1;13:18. doi: 10.1186/s40621-026-00659-4 (PMC12952156; doi:10.1186/s40621-026-00659-4)
Supplement: Supplementary file 3 — Supplementary Material 3 [file 40621_2026_659_MOESM3_ESM.docx]

**Supplemental material for *Examining disparities in harmful reporting on community firearm violence in television news reports***

**Appendix C.** Variable and corresponding data sources, constructs, and descriptions

| **Variable type** | **Source** | **Name of variable / category** | **Description (how variable was modeled)** |
| --- | --- | --- | --- |
| Dependent variables | Coded TV news clips (n=303) | Harmful CFV reporting score | - Summed score based on presence or absence of harmful reporting elements each multiplied by severity of harm assigned from Delphi panel study, constructed at the individual, community, and society levels (continuous) |
|  |  | Harmful CFV reporting elements | - Presence or absence of each of the 11 harmful reporting elements, which were: graphic and/or explicit content; clinical condition; number of gunshot wounds; name of the treating hospital; relationship between the firearm-injured person and the perpetrator; mugshot of perpetrator; overall episodic framing; only law enforcement narrators, missing perspective of firearm-injured person; and missing community perspective (binary) |
| Independent variables | Philadelphia Police Department data | Victim characteristics | - Race/ethnicity as Black, Hispanic, Asian, or white (categorical with reference group of Black) - Age as 18 and older or under 18 (categorical with reference group of 18 and older) - Sex as either male or female (categorical with reference group of male) |
|  |  | Event characteristics | - Fatal shooting as any time any person injured in the shooting was recorded as having died, non-fatal otherwise (categorical with reference group of non-fatal) - Mass shooting as any time four or more people were injured or killed in a single shooting event and non-mass shooting otherwise (categorical with reference group of non-mass shooting) |
|  | American Community Survey 5-year estimates for 2015-2019 | Place-based characteristics | - Percent Black residents as quintiles of the percentage of Black residents in the census block group where the shooting occurred (continuous) - Percent of residents unemployed as quintiles of the percentage of residents who are unemployed in the census block group where the shooting occurred (continuous) - Median household income as quintiles of the median household income in US dollars in the census tract where the shooting occurred (continuous) - Percent poverty as quintiles of the percentage of residents with income under the US Census Bureau poverty thresholds in the census tract where the shooting occurred (continuous) - Income inequality as quintiles of the Gini coefficient calculated for the census tract where the shooting occurred (continuous) - Racialized economic segregation as quintiles of the Index of Concentration at the Extremes for race and income calculated for the census tract where the shooting occurred; higher scores indicate greater concentrated advantage (continuous) |
|  | Coded TV news clips | News coverage characteristics | - More than one clip as any time the individual had more than one clip included in the content analysis dataset, one clip otherwise (categorical with reference group of one clip) - Total clip length as quintiles of the total number of seconds of all clips for each individual (continuous) - Total focus time as quintiles of the total number of seconds focused primarily on the shooting event of all clips for each individual (continuous) - Include follow-up story as any time any of the clips for an individual in the content analysis dataset was a follow-up story, does not include a follow-up story otherwise (categorical with reference group of does not include a follow-up story) |
