# Supplementary material for: Examining disparities in harmful reporting on community firearm violence in Philadelphia television news reports
Source: Inj Epidemiol. 2026 Feb 1;13:18. doi: 10.1186/s40621-026-00659-4 (PMC12952156; doi:10.1186/s40621-026-00659-4)
Supplement: Supplementary file 4 — Supplementary Material 4 [file 40621_2026_659_MOESM4_ESM.docx]

**Supplemental material for *Examining disparities in harmful reporting on community firearm violence in television news reports***

**Appendix D.** Pairwise correlation matrix for the presence/absence of harmful reporting elements

|  | 1 | 2 | 3 | 4 | 5 | 6 | 7 | 8 | 9 | 10 | 11 |
| --- | --- | --- | --- | --- | --- | --- | --- | --- | --- | --- | --- |
| 1. Overall episodic framing | 1 | - | - | - | - | - | - | - | - | - | - |
| 2. Missing perspective of firearm-injured person | -0.01  *p* = .855 | 1 | - | - | - | - | - | - | - | - | - |
| 3. Does not cover solutions | **0.39**  ***p* < .001** | **0.25**  ***p* < .001** | 1 | - | - | - | - | - | - | - | - |
| 4. Missing community perspective | **0.18**  ***p* < .001** | **0.20**  ***p* < .001** | **0.43**  ***p* < .001** | 1 | - | - | - | - | - | - | - |
| 5. Number of gunshot wounds | 0.09  *p* = .084 | 0.002  *p* = .97 | -0.01  *p* = .824 | **-0.17**  ***p* < .001** | 1 | - | - | - | - | - | - |
| 6. Clinical condition of firearm-injured person | 0.09  *p* = .068 | **0.27**  ***p* < .001** | 0.04  *p* = .484 | 0.07  *p* = .181 | 0.01  *p* = .850 | 1 | - | - | - | - | - |
| 7. Only law enforcement narrators | **0.18**  ***p* < .001** | **0.23**  ***p* < .001** | **0.40**  ***p* < .001** | **0.43**  ***p* < .001** | -0.04  *p* = .453 | -0.04  *p* = .462 | 1 | - | - | - | - |
| 8. Graphic and/or explicit content | -0.07  *p* = .197 | -0.02  *p* = .730 | **-0.34**  ***p* < .001** | **-0.34**  ***p* < .001** | 0.10  *p* = .059 | 0.09  *p* = .077 | **-0.23**  ***p* < .001** | 1 | - | - | - |
| 9. Name of treating hospital | **0.14**  ***p* < .01** | 0.03  *p* = .528 | **0.23**  ***p* < .001** | 0.03  *p* = .585 | **0.13**  ***p* = .02** | **0.27**  ***p* < .001** | 0.07  *p* = .164 | -0.06  *p* = .268 | 1 | - | - |
| 10. Mugshot of perpetrator | 0.06  *p* = .256 | -0.09  *p* = .074 | **-0.11**  ***p* = .026** | -0.08  *p* = .112 | 0.08  *p* = .128 | **-0.13**  ***p* = .011** | -0.06  *p* = .240 | 0.09  *p* = .090 | -0.10  *p* = .059 | 1 | - |
| 11. Relationship between firearm-injured person and perpetrator | 0.06  *p* = .256 | -0.05  *p* = .328 | -0.004  *p* = .945 | **-0.11**  ***p* = .034** | 0.08  *p* = .128 | -0.06  *p* = .275 | -0.08  *p* = .094 | -0.04  *p* = .387 | 0.07  *p* = .146 | **0.28**  ***p* < .001** | 1 |

*Notes.* Reported *φ* values represent the correlation between the presence (1) or absence (0) of harmful reporting elements among individuals (*N* = 394) covered in a random selection of Philadelphia TV news clips, 2021. Elements are ordered by frequency. Bolded values indicate *p* < .05.
