# Supplementary material for: Examining disparities in harmful reporting on community firearm violence in Philadelphia television news reports
Source: Inj Epidemiol. 2026 Feb 1;13:18. doi: 10.1186/s40621-026-00659-4 (PMC12952156; doi:10.1186/s40621-026-00659-4)
Supplement: Supplementary file 5 — Supplementary Material 5 [file 40621_2026_659_MOESM5_ESM.docx]

**Supplemental material for *Examining disparities in harmful reporting on community firearm violence in television news reports***

**Appendix E.** Direct paths from victim, event, place-based, and coverage characteristics to presence of each harmful community firearm violence reporting content element

|  | **Harmful community firearm violence reporting content elements** | | | | | | | | | | | | | | | | | |
| --- | --- | --- | --- | --- | --- | --- | --- | --- | --- | --- | --- | --- | --- | --- | --- | --- | --- | --- |
|  | Graphic and/or explicit content | | Clinical condition | | Number of gunshot wounds | | Name of treating hospital | | Relationship | | Mugshot of perpetrator | | Only law enforcement narrators | | Missing community perspective | | Does not explore solutions | |
| **Independent variable** | B | SE | B | SE | B | SE | B | SE | B | SE | B | SE | B | SE | B | SE | B | SE |
| ***Victim characteristics*** | | | | | | | | | | | | | | | | | | |
| Race/ethnicity |  |  |  |  |  |  |  |  |  |  |  |  |  |  |  |  |  |  |
| Black | Ref. | - | Ref. | - | Ref. | - | Ref. | - | Ref. | - | Ref. | - | Ref. | - | Ref. | - | Ref. | - |
| Hispanic | -0.25 | 0.52 | -0.80 | 0.52 | 0.22 | 0.47 | 0.28 | 0.57 | 0.14 | 1.07 | 1.74 | 0.91 | 1.07 | 0.56 | 1.13 | 0.64 | 0.41 | 0.71 |
| Asian | ‡ | ‡ | ‡ | ‡ | ‡ | ‡ | ‡ | ‡ | ‡ | ‡ | ‡ | ‡ | ‡ | ‡ | ‡ | ‡ | ‡ | ‡ |
| White | **-1.23*** | **0.55** | -0.89 | 0.46 | 0.57 | 0.44 | 0.88 | 0.45 | 0.98 | 0.77 | **2.76*** | **1.12** | **0.98*** | **0.46** | 0.26 | 0.57 | 0.04 | 0.71 |
| Age |  |  |  |  |  |  |  |  |  |  |  |  |  |  |  |  |  |  |
| 18 and older | Ref. | - | Ref. | - | Ref. | - | Ref. | - | Ref. | - | Ref. | - | Ref. | - | Ref. | - | Ref. | - |
| Under 18 | **-0.81** | **0.36** | 0.43 | 0.36 | 0.21 | 0.31 | **0.70*** | **0.33** | 0.98 | 0.77 | 0.12 | 0.88 | -0.04 | 0.38 | 0.45 | 0.40 | **-1.28**** | **0.44** |
| Sex |  |  |  |  |  |  |  |  |  |  |  |  |  |  |  |  |  |  |
| Male | Ref. | - | Ref. | - | Ref. | - | Ref. | - | Ref. | - | Ref. | - | Ref. | - | Ref. | - | Ref. | - |
| Female | -0.63 | 0.37 | -0.06 | 0.37 | 0.19 | 0.31 | -0.48 | 0.39 | **3.15***** | **0.75** | 1.14 | 0.65 | -0.32 | 0.39 | 0.69 | 0.42 | 0.95 | 0.49 |
| ***Event characteristics*** | | | | | | | | | | | | | | | | | | |
| Fatal shooting |  |  |  |  |  |  |  |  |  |  |  |  |  |  |  |  |  |  |
| Non-fatal | Ref. | - | Ref. | - | Ref. | - | Ref. | - | Ref. | - | Ref. | - | Ref. | - | Ref. | - | Ref. | - |
| Fatal | **-0.85**** | **0.32** | **-2.44***** | **0.31** | -0.11 | 0.25 | **-0.80**** | **0.29** | -0.40 | 0.82 | 1.98 | 1.15 | -0.47 | 0.29 | -0.20 | 0.37 | 0.21 | 0.42 |
| Mass shooting |  |  |  |  |  |  |  |  |  |  |  |  |  |  |  |  |  |  |
| Not a mass shooting | Ref. | - | Ref. | - | Ref. | - | Ref. | - | Ref. | - | Ref. | - | Ref. | - | Ref. | - | Ref. | - |
| Mass shooting | -0.38 | 0.45 | **1.10*** | **0.38** | **-1.27***** | **0.25** | -0.75 | 0.48 | ‡ | ‡ | ‡ | ‡ | 0.01 | 0.54 | **1.31*** | **0.53** | -0.02 | 0.57 |
| ***Place-based characteristics*** | | | | | | | | | | | | | | | | | | |
| Percent Black residents (CBG) | **0.39**** | **0.14** | **-0.32*** | **0.14** | **0.29*** | **0.12** | 0.12 | 0.14 | 0.13 | 0.31 | -0.46 | 0.40 | -0.10 | 0.14 | **-0.51**** | **0.17** | **-0.43*** | **0.19** |
| Percent of residents unemployed (CBG) | -0.02 | 0.82 | -0.17 | 0.09 | 0.06 | 0.08 | -0.09 | 0.10 | **-0.66*** | **0.48** | -0.31 | 0.25 | 0.16 | 0.10 | **0.25*** | **0.12** | **-0.36**** | **0.14** |
| Median household income (CT) | -0.23 | 0.19 | 0.31 | 0.18 | 0.24 | 0.15 | **0.84***** | **0.19** | -0.69 | 0.48 | 0.18 | 0.25 | -0.06 | 0.19 | 0.19 | 0.22 | **0.58**** | **0.26** |
| Percent poverty (CT) | 0.15 | 0.16 | -0.16 | 0.15 | -0.01 | 0.13 | **0.53***** | **0.16** | -0.21 | 0.44 | 0.30 | 0.40 | -0.03 | 0.16 | -0.11 | 0.18 | 0.22 | 0.21 |
| Income inequality (CT) | 0.06 | 0.12 | 0.16 | 0.11 | 0.08 | 0.09 | -0.16 | 0.12 | -0.64 | 0.33 | **-0.60*** | **0.29** | 0.12 | 0.11 | -0.08 | 0.14 | -0.30 | 0.16 |
| Racialized economic segregation (CT) | **0.85***** | **0.21** | **-0.57**** | **0.19** | 0.19 | 0.16 | **-0.53**** | **0.19** | 0.27 | 0.45 | -0.73 | 0.47 | -0.08 | 0.18 | **-0.87***** | **0.24** | **-0.77**** | **0.28** |
| ***Coverage characteristics*** | | | | | | | | | | | | | | | | | | |
| Number of clips |  |  |  |  |  |  |  |  |  |  |  |  |  |  |  |  |  |  |
| One clip | Ref. | - | Ref. | - | Ref. | - | Ref. | - | Ref. | - | Ref. | - | Ref. | - | Ref. | - | Ref. | - |
| More than one clip | **0.95*** | **0.37** | **1.45***** | **0.42** | **0.89*** | **0.37** | 0.59 | 0.43 | -0.62 | 0.79 | 1.42 | 0.83 | 0.37 | 0.53 | 0.53 | 0.40 | -0.47 | 0.44 |
| Total clip length | 0.36 | 0.22 | **-0.53*** | **0.24** | 0.09 | 0.19 | **-0.55*** | **0.26** | 0.41 | 0.65 | -0.90 | 0.83 | **-0.64***** | **0.22** | **-1.64***** | **0.27** | **-2.07*** | **0.32** |
| Total focus time | 0.14 | 0.21 | **0.87***** | **0.24** | 0.14 | 0.19 | **0.71**** | **0.25** | 1.13 | 0.66 | 1.21 | 0.82 | -0.28 | 0.22 | 0.29 | 0.24 | **0.61*** | **0.27** |
| Follow-up story |  |  |  |  |  |  |  |  |  |  |  |  |  |  |  |  |  |  |
| Does not include follow-  up story | Ref. | - | Ref. | - | Ref. | - | Ref. | - | Ref. | - | Ref. | - | Ref. | - | Ref. | - | Ref. | - |
| Includes follow-up story | **1.15**** | **0.40** | **-1.43***** | **0.43** | **-0.82*** | **0.35** | -0.80 | 0.42 | 0.54 | 0.92 | ‡ | ‡ | **-1.07*** | **0.43** | **-1.09*** | **0.43** | **-1.06*** | **0.48** |

*Notes*. Unstandardized estimates from generalized structural equation models examining relationships from demographic, event, place-based, and coverage characteristics to harmful gun violence reporting scale scores. Bolded values indicate *p* < .05.
CBG = census block group; CT = census tract
* = *p* < .05; ** = *p* < .01; *** = *p* < .001; ‡ = Suppressed due to unreliability of estimate
